# Identification and characterization of the microRNA transcriptome of a moth orchid *Phalaenopsis aphrodite*

**DOI:** 10.1007/s11103-013-0150-0

**Published:** 2013-10-31

**Authors:** Ya-Ting Chao, Chun-Lin Su, Wen-Han Jean, Wan-Chieh Chen, Yao-Chien Alex Chang, Ming-Che Shih

**Affiliations:** 1 Agricultural Biotechnology Research Center, Academia Sinica, Taipei, 11529 Taiwan; 2 Department of Horticulture and Landscape Architecture, National Taiwan University, Taipei, 10617 Taiwan

**Keywords:** *Phalaenopsis aphrodite*, Orchid, Non-model plants, microRNA, Deep sequencing, Bioinformatics

## Abstract

Orchids display unique phenotypes, functional characteristics and ecological adaptations that are not found in model plants. In this study, we aimed to characterize the microRNA (miRNA) transcriptome and identify species- and tissue-specific miRNAs in *Phalaenopsis aphrodite*. After data filtering and cleanup, a total of 59,387,374 reads, representing 1,649,996 unique reads, were obtained from four *P. aphrodite* small RNA libraries. A systematic bioinformatics analysis pipeline was developed that can be used for miRNA and precursor mining, and target gene prediction in non-model plants. A total of 3,251 unique reads for 181 known plant miRNAs (belonging to 88 miRNA families), 23 new miRNAs and 91 precursors were identified. All the miRNA star sequences (miRNA*), the complementary strands of miRNA that from miRNA/miRNA* duplexes, of the predicted new miRNAs were detected in our small RNA libraries, providing additional evidence for their existence as new miRNAs in *P. aphrodite*. Furthermore, 240 potential miRNA-targets that appear to be involved in many different biological activities and molecular functions, especially transcription factors, were identified, suggesting that miRNAs can impact multiple processes in *P. aphrodite*. We also verified the cleavage sites for six targets using RNA ligase-mediated rapid amplification of 5′ ends assay. The results provide valuable information about the composition, expression and function of miRNA in *P. aphrodite*, and will aid functional genomics studies of orchids.

**Electronic supplementary material:**

The online version of this article (doi:10.1007/s11103-013-0150-0) contains supplementary material, which is available to authorized users.

## Introduction

The Orchidaceae is the largest family of angiosperms with 25,000–30,000 species widely distributed around the world (Pridgeon et al. 2005). The unique and exotic floral organs of orchids have made them a popular commercial crop with considerable economic value. In addition to their ornamental value, orchids display several fascinating biological and ecological features that are of particular interest to plant biologists. Orchids exhibit floral bilateral symmetry (zygomorphy) in contrast to the radial symmetry (actinomorphy) seen in most flowers. Their floral organs are arranged in unique patterns such as perianths with sepals and petals of a similar shape and color (termed “tepals”), pistil and stamen fusion, and enlargement of one petal into an extravagant “lip” structure (Rudall and Bateman 2002). Some orchids, such as *Phalaenopsis*, are epiphytic plants with high water-use efficiency. They carry out crassulacean acid metabolism (CAM) photosynthesis assimilating carbon dioxide at night (Guo and Lee 2006; Silvera et al. 2010). Orchids also exhibit unique seed development characteristics such as lacking endosperms (or cotyledons) for nutrient supply and having embryos that stall at the globular stage until germination (Vinogradova and Andronova 2002). These unusual traits make orchids attractive research objects through which to explore some of the more diverse plant phenomena not seen in model organisms such as Arabidopsis and rice. Our group is particularly interested in the analysis of a category of small RNA, microRNA (miRNA). We hope that analysis of miRNA will further our understanding of the gene expression of orchids in various tissues or at different developmental stages. To the best of our knowledge, only one study has previously investigated miRNA in *Phalaenopsis orchid* (An et al. 2011). Orchid miRNAs and their targets still remain largely unknown. In this study we have developed a bioinformatics pipeline for the analysis of known and new miRNAs and their targets in *P. aphrodite*, and constructed a web-based orchid miRNA database.

miRNAs are endogenous small non-coding RNAs that have been demonstrated to play a crucial role in post-transcriptional regulation (for reviews, see (Filipowicz et al. 2008; Voinnet 2009). miRNA genes are generally transcribed by RNA polymerase II to produce primary miRNAs (pri-miRNAs) that contain internal stem-loop regions. In plants, pri-miRNA is processed into a stem-loop precursor (pre-miRNA) by the proteins Dicer-like 1 (DCL1) and Hyponastic Leaves 1 (HYL1), and further cleaved by DCL1 to release a ~22 bp miRNA duplex (miRNA/miRNA*) with 2 nt overhangs at the 3′ ends (Voinnet 2009). The miRNA duplex then separates into a biologically active strand (miRNA) and a passenger strand (miRNA*, the complementary strand of miRNA). miRNA executes its regulatory function through binding to the complementary site on its target mRNAs to induce transcript cleavage or translational repression.

Several different approaches, such as direct cloning, northern blotting, stem-loop real-time RT-PCR and microarray technologies, are commonly used to detect and identify specific miRNAs under various treatment conditions. Recently, deep sequencing technology has been demonstrated to be an effective method for miRNA discovery and profiling in model organisms (Sunkar et al. 2008; Wang et al. 2011; Kato et al. 2009; Zhang et al. 2009a) and many other genomes (Gonzalez-Ibeas et al. 2011; Song et al. 2010; Lelandais-Briere et al. 2009; Morin et al. 2008; Bar et al. 2008; Xia et al. 2012). However, the vast amounts of data obtained from deep sequencing pose challenges in efficient and reliable discovery of new miRNA. Moreover, distinguishing heterozygous sequence variants from sequencing errors could be more challenging in non-model plants, due to the lack of genome sequences for mapping/aligning.

Many computational tools have been developed to facilitate systematic prediction of miRNA and pre-miRNA. Previous studies have revealed that some miRNA families are widely conserved across the plant lineages such as mosses, gymnosperms, monocots and eudicots (Zhang et al. 2006; Axtell and Bartel 2005), indicating that computer-based homology search should provide a powerful strategy for the discovery and identification of mature sequences of conserved miRNA families. The characteristic stem-loop pre-miRNA structure and the high degree of conservation of mature sequences between related genomes are important features of miRNA genes that are exploited in their computational identification (Lim et al. 2003; Lai et al. 2003; Dezulian et al. 2006; Huang et al. 2007). In *Arabidopsis thaliana* and rice genomes, comparative genomics-based methods have been used to identify highly conserved families of miRNA and their targets (Bonnet et al. 2004; Jones-Rhoades and Bartel 2004; Wang et al. 2004). However, homology search-based methods are not applicable to the detection of species-specific miRNAs, or the detection of plant pre-miRNA.

In Arabidopsis, a single genome-based analysis was performed using the findMiRNA algorithm to detect miRNAs (Adai et al. 2005). The findMiRNA algorithm uses Arabidopsis transcript sequences and looks for corresponding short sequences embedded in intergenic- or intron-hairpins within candidate miRNA precursors (pre-miRNAs) that have the potential to target any part of these transcripts (Adai et al. 2005). Since miRNA precursors are transcribed by RNA polymerase II and polyadenylated (Lee et al. 2004), some pre-miRNAs should be represented by expressed sequence tags (EST). EST analysis (Zhang et al. 2005) is widely used in model plants and species with limited genomic resources (Han et al. 2010; Colaiacovo et al. 2010; Bhardwaj et al. 2010; Kim et al. 2011). The EST approach is usually based on a sequence similarity search step followed by a set of structural filters. Plant pre-miRNA identification is more difficult than animal pre-miRNA prediction because plant pre-miRNA stem-loops differ greatly in size and structure. Several machine-learning based prediction programs have been designed to distinguish real pre-miRNAs from other hairpin sequences with similar stem-loops (Jiang et al. 2007; Huang et al. 2007; Xuan et al. 2011; Xue et al. 2005). Mirroring plant miRNA gene prediction studies, the algorithms for predicting plant miRNA target have largely focused on the model Arabidopsis and rice genomes (Wang et al. 2004; Rhoades et al. 2002; Zhang 2005).

Here we applied next generation sequencing (NGS) technology to investigate the small RNA transcriptome of the moth orchid *Phalaenopsis aphrodite*. An informatics pipeline was designed to optimize the analysis of sequence outputs collected from the Illumina genome analyser. Both known and novel miRNAs and non-coding transcripts that represent the corresponding miRNA precursors were identified in *P. aphrodite*. The expression profiles of the miRNAs in various tissues were verified by stem-loop real-time RT-PCR, and miRNA-target gene prediction was performed using both homology-dependent and homology-independent methods. In addition, six target genes were experimentally verified. The results were integrated into a web-based orchid database named Orchidstra (http://orchidstra.abrc.sinica.edu.tw).

## Materials and methods

### Plant materials and RNA isolation

Taiwan endemic moth orchid, *P. aphrodite* Rchb.f. collected from its native mountain habitat in Dawu, Taitung County, was kindly provided by Dr. Tsai-Mu Shen from National Chiayi University, Chiayi County, Taiwan. Mature plants were maintained in 22–27 °C growth chambers under a 12-h day/night light cycle with regular irrigation and fertilization. Seeds from hand-pollinated capsules (120 days after pollination) were germinated on one-fourth Murashige and Skoog medium supplemented with Gamborg B_5_ vitamins (Duchefa Biochemie, Netherlands), 1 % tryptone, 2 % sucrose and 0.85 % agar at pH 5.6 under the same growth conditions as the mature plants. Four orchid small RNA libraries were constructed from a collection of various orchid tissues including mature leaves, roots, flowers, and germinating seeds of multiple, randomly selected plants. A flower library was built by pooling tissues of young inflorescences with mature flower buds and flowers in full bloom. The seed library was constructed by randomly collecting germinating seeds at various stages, including the protocorm formation stage [0–30 days after sowing (DAS)], the protocorm development stage (40–75 DAS), and the seedling formation stage (75–100 DAS). Total RNA was isolated as previously described (Su et al. 2011) and quality was confirmed using RNA Bioanalyzer (Agilent, CA, USA). We used 10 μg total RNA as the initial input for library construction.

### Illumina cDNA library preparation

Massively parallel sequencing was performed on the Illumina Genome Analyzer IIx system. Small RNA (18–30 bp) was gel-purified by 6 % Novex TBE polyacrylamid gel electrophoresis (Invitrogen, CA, USA) followed by gel elution according to the supplier’s protocol. All libraries were constructed using Small RNA Sample Preparation Kits (Illumina, CA, USA) and included 5′ and 3′ RNA adaptor ligation, reverse transcription, PCR amplification and purification. Single-end sequencing of the cDNA libraries was then performed using Illumina. All procedures followed the protocols provided by the manufacturer. A total of 97,147,780 (23,852,494 reads for root, 24,059,282 for leaf, 23,741,532 for flower, and 25,494,472 for seed) 40-bp reads were generated (GenBank: SRA050114). Illumina sequencing was not replicated.

### Bioinformatic analysis of small RNA deep sequencing data

**Fig. 1:**
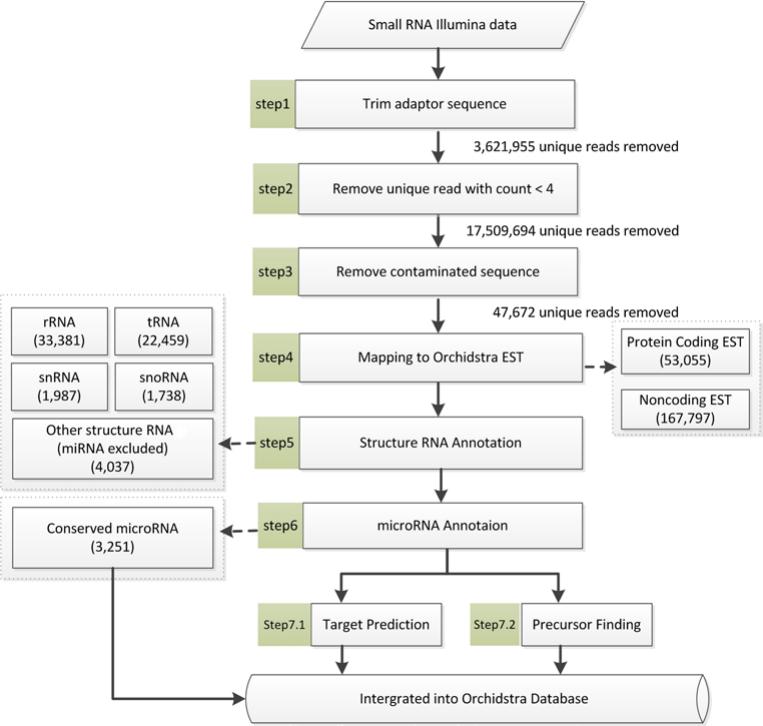
Annotation pipeline for small RNA reads and summary of results. Numbers in *parentheses* indicate the number of unique reads across all libraries. See Table 2 for the number of unique reads and total reads in each library

Sequencing data processing and small RNA analysis pipeline is illustrated in Fig. 1. The small RNA data output from Illumina was processed with in-house programs to collapse identical reads into a single read (a unique read) while recording the number of times that unique read was observed in each library. Only the reads that were completely identical in both length and sequence were collapsed into a unique read. Reads with a difference even in only one nucleotide were not merged together, ensuring that the data processing did not eliminate any heterozygosity in the sequences. If a read contains a low quality segment (most bases have quality values of Q15 or below), the base calls of the segment were perceived as unreliable and all of the quality values in the segment were replaced with a value of 2 by the base-calling program, and the corresponding bases were represented by Ns in the read (CASAVA Software Version 1.7 User Guide). Such reads containing poly-N segment were excluded in further analysis in this study. The data were converted to an acceptable format for DSAP (Huang et al. 2010) to remove adapter sequences and poly-A/T/C/G/N nucleotides. The sequence reads were further filtered to remove reads appearing less than four times in total across the libraries. It has been reported that the primary errors in reads from Illumina sequencers are substitution errors, reads with very low coverage usually represent sequencing errors (Kelley et al. 2010). Filtering out all reads with counts less than a low threshold is a strategy used to eliminate sequencing errors (Motameny et al. 2010) that is of critical importance for distinguishing heterozygous variants from artefacts of sequencing errors when a reference genome is not available. Because the sequencing achieved a sufficient depth in this study, our aim was to extract useful informative reads that are distinguishable from the artefacts caused by sequencing errors. The reads that were removed by the low-count filter were considered to be unreliable and uninformative. After low-count filtering, the remaining reads were also considered to show evidence of expression at a meaningful level. Next, several databases were retrieved for further processing. The sequences from the chloroplast genome of *P. aphrodite*, orchid pathogens including *Cymbidium mosaic virus* and *Odontoglossum ringspot virus*, and *Escherichia coli* were downloaded from the NCBI database; 42,590 protein-coding *P. aphrodite* ESTs and 191,263 unknown/non-coding *P. aphrodite* ESTs were obtained from a previous study (Su et al. 2011). The small RNA unique reads from quality-trimming and filtering were BLAST searched against the orchid chloroplast, the virus sequences and *E. coli* sequences. BLAST search was performed with default setting, and then the perfect matches (alignments have 100 % identity and span the whole length of the query read) were parsed out from the raw BLAST output. The remaining unique reads were then BLASTN searched against the Orchidstra database. Because the protein-coding ESTs in the Orchidstra database were in the orientation of the coding strand and our small RNA sequencing is strand-specific, the unique reads that aligned perfectly with the protein-coding ESTs in the same orientation were considered to be degraded mRNA and removed from further analysis.

RNA family data and known miRNA sequences were downloaded from Rfam version 10 and miRBase version 18. To annotate sequences related to structural RNA, we ran BLASTN to search against the Rfam database with default setting, and parsed out the alignments with up to three discrepancies (gaps plus mismatches) from the raw BLAST output. All unique reads left from the previous screening were searched against the miRBase using BLASTN with same parameters values that the miRBase Web-Blast server uses (–W 4 –r 5 –q −4). Unique reads that were identical to or had less than three mismatches to known miRNAs were regarded as potential conserved miRNAs in *P. aphrodite*.

### Statistical analysis and the specificity measure of miRNA expression

The counts of unique reads were normalized to reads per million (RPM) by dividing the raw read count by the total number of reads in each library and multiplying by one million. The expression profiles for each miRNA family were calculated by summing all reads annotated to the same miRNA family in each library. The RPM values of each miRNA family were log-transformed to base 2 and imported into GeneSpring 11.5.1 software (Agilent, CA, USA) to perform cluster analysis using a Euclidean distance matrix and the centroid linkage rule. The specificity measure (SPM) of each miRNA was calculated using the method described in (Xiao et al. 2010). Statistical analysis was performed using the stats package in R version 2.15 (R Development Core Team 2012).

### miRNA precursor prediction procedure

To screen miRNA precursors from non-coding ESTs, we checked (1) whether a putative mature miRNA was present in the stem and not in the loop of the stem-loop structure of the non-coding EST and (2) whether the stem-loop structure was similar to the structure of known miRNAs in miRBase. The following non-coding Orchidstra ESTs were sent to the precursor prediction pipeline to examine their secondary structures: non-coding ESTs with a perfect match to *P. aphrodite* conserved miRNAs, non-coding ESTs with a perfect match to unknown *P. aphrodite* small RNA reads, and non-coding EST with 3 or fewer mismatches (gaps plus mismatches) to miRBase registered miRNAs. Supplemental Figure 1 illustrates a schematic overview of the process by which miRNA precursors were searched for in *P. aphrodite*. The RNA secondary structures of both the forward and the reverse complement of the non-coding ESTs were predicted using Mfold software (Zuker 2003). Folding temperature was set at 25 °C and the default settings were used for the other parameters. The resulting secondary structures were checked for the location of putative mature miRNA, which had to be fully contained in a double stranded region of the hairpin structure. Since the stem is slightly longer than the length of the mature miRNA, the sequences located 10 nt outside the terminal basepair between the miRNA and miRNA* were trimmed off, and the resulting sequences were recalculated for secondary structure and minimum free energy (MFE). In order to define the structural features filter, we established criteria that accounted for matches, mismatches (bulges) and gaps on the stem region, the occurrence of multi-loops, as well as MFE. The criteria were chosen based on the statistics for the predicted stem-loop structures (including statistics on number/size of matches, gaps, bulges and loops) of the known pre-miRNA sequences in the miRBase (data not shown). The sequences were not the same length, thus their MFEs are not directly comparable. Instead of using an arbitrary chosen MFE threshold for all sequences, in order to determine an adequate MFE filter, the MFE of experimentally validated miRNAs was regressed on the sequence length to construct the 95 % prediction interval for MFE. stem-loop structures with MFE falling outside the prediction interval were discarded. We defined the stem containing EST residues that aligned with small RNA/miRNA as the “core region” of a precursor candidate. After the MFE filter, further evaluation of the precursor candidates took into account the size of loop (with a maximum cut-off of 15 nt), the branch number (with a maximum cut-off of 6), the bulge number in the core region (maximum 1 allowed), the bulge size in the core region (maximum 2 allowed), the number of continuous unpaired residues (maximum 4 allowed) in the core region, and no loop/branch inside the core region. The number of allowed mismatches varied with the length of the core region. For a core region of length up to 17 bp, the maximum number of unpaired residues was 1. The maximum number of unpaired residues allowed for cores with lengths of 18, 19, 20, and over 20 bp was 2, 3, 4, and 5, respectively. A non-coding EST was considered a miRNA precursor if it fulfilled the specified criteria. All the predicted precursors have a characteristic stem-loop structure with the mature miRNA embedded in the arm of the stem.

To assess the sensitivity and specificity of our precursor prediction procedure, we need to access the quantity of true positives, true negatives, false positives and false negatives, thus we used Arabidopsis genome, the most well-annotated plant genome, to calculate the sensitivity and specificity. We downloaded 328 Arabidopsis pre-miRNA sequences in miRBase using them as positive examples, and also generated 328 negative examples by permuting each pre-miRNA sequence while preserving its nucleotide frequencies. Our pre-miRNA prediction pipeline achieved good accuracy with a sensitivity of 85 % (278/328) and a specificity of 100.0 %.

### Target prediction procedure

Two computational approaches were applied to predict miRNA target transcripts in this study (Supplemental Figure 2a). One approach is based on the observed property of extensive complementarity between plant miRNAs and their targets (Axtell and Bowman 2008). miRNAs were searched against the protein-coding ESTs in Orchidstra with -g (gapped alignment) F (false) appended for BLASTN to prevent the insertion of gaps in the middle of alignment. To filter out miRNA target candidates from BLASTN results, the antisense hits (alignments with complementary matches) were checked for the number of mismatches and alignment length. In this procedure, only Watson–Crick base pairing was allowed; G:U pairs were not considered to be a match. Alignments containing positions 2–12 of the miRNA with an alignment length over 16 nt and three or less mismatches were considered to be miRNA target candidates. This prediction procedure was similar to a previous study (Rhoades et al. 2002).

In the second procedure protein-coding ESTs in Orchidstra were blastx searched against the TAIR10 database (http://www.arabidopsis.org/index.jsp) using a cutoff value of 1e-30. An EST was considered a candidate miRNA target if its best BLAST match was a target gene of an miRNA listed in the Arabidopsis small RNA project (ASRP) database (http://asrp.cgrb.oregonstate.edu/) (Gustafson et al. 2005).

### Stem-loop RT-PCR of miRNAs and RT-qPCR of targets

Tissues from multiple plants were pooled by tissue types. The cDNA of the mature miRNA was prepared using a Taqman MicroRNA reverse transcription (RT) kit (ABI, 4366596) according to the manufacturer’s protocol. Stem-loop RT primers and forward qPCR primers were designed according to the rules described in (Chen et al. 2005). The sequences of the primers are listed in Supplemental Table 1. One microliter of 3× diluted first strand cDNA solution was used as the template for subsequent PCR amplification. Real-time PCR was performed using SYBR Green PCR Master Mix and the Applied Biosystems 7300 Real-Time PCR System. The PCR reactions were performed at 95 °C for 10 min, followed by 40 cycles of 95 °C for 15 s and 60 °C for 1 min. The expression levels of each mature miRNA were recorded by the threshold cycle (Ct) and normalized against the internal control (PASR17041531, annotated as miR5139). The transcript levels of each target gene were detected by RT-qPCR analysis, the actin gene (PATC135993) was amplified as a reference using the primer pair, forward primer: 5′-CTAGCGGAAACGCGACAGA and reverse primer: 5′-CCAAGGGAAGCCAAAATGC. Three technical replications were performed for each miRNA/target gene in each tissue type for qRT-PCR. Three biological replications were performed for qRT-PCR of miRNA using leaves and roots from individual plants.

### Experimental validation of selected miRNA targets

In order to identify the cleavage sites within the miRNA targets, we performed the RNA ligase-mediated rapid amplification of 5′ ends (RLM-5′ RACE) experiment using the GeneRacer Kit (Invitrogen Life Technologies, CA, USA) according to the manufacturer’s instructions and (Llave et al. 2002). Total RNA from roots, leaves and flowers was isolated as previously described (Su et al. 2011) and mRNA was extracted from the total RNA using PolyATtract mRNA Isolation System (Promega). Briefly, the RNA adapter (Supplemental Table 2) was ligated to the 5′ ends of mRNA without enzymatic pretreatment. The ligated products were reverse transcribed and PCR amplified with gene specific primers (GSP) listed in Supplemental Table 2. The 5′ RACE-PCR products were cloned into pZeroBack vector (Tiangen Biotech Co., China). Ten to twenty-five positive clones were picked and sequenced for each target gene.

## Results and discussion

### Analysis and annotation of small RNA deep sequencing data

**Fig. 2:**
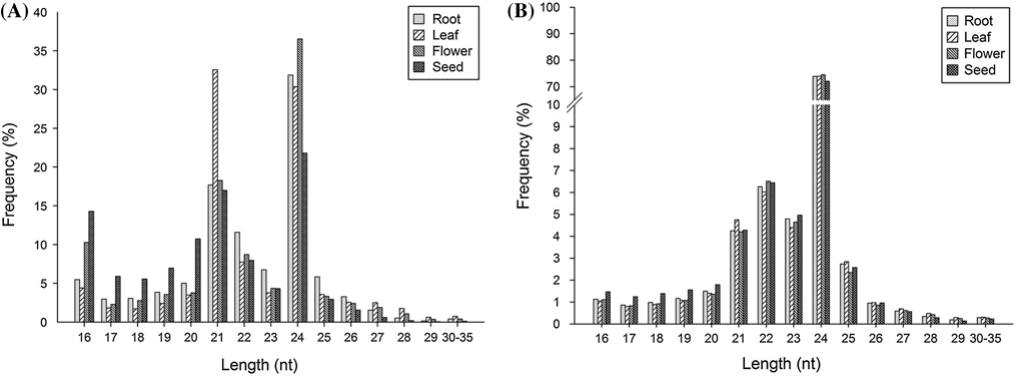
Summary of next generation sequencing data of the small RNA transcriptome of *P. aphrodite*. **a** Read-length distribution after removing poly-A/T/C/G/N nucleotides and trimming the adapter sequences. **b** Length distribution of unique reads

In order to identify conserved miRNAs and novel miRNAs in *P. aphrodite*, small RNA transcriptomes from root, leaf, flower and seed libraries were sequenced on Illumina Genome Analyzer II. A total of 83,942,304 sequencing reads were obtained after removing adapter sequences and poly-A/T/C/G/N nucleotides (see “Materials and methods” and Table 1). The cleaned sequence reads were grouped into unique sequence reads. Unique reads were filtered out if they appeared less than four times in total across the libraries. The remaining sequences that mapped to the chloroplast or virus genomes (see “Materials and methods” section) were then discarded. After data cleanup and filtering, there were 1,164,475, 1,117,720, 1,206,840 and 1,047,951 unique reads in the root, leaf, flower and seed libraries, respectively (Table 1). The lengths of small RNA reads ranged from 16 nt to over 30 nt, with the majority being 21 nt and 24 nt (Fig. 2a). The length distribution plot of unique small RNAs had only one major peak at 24 nt (Fig. 2b), indicating that the 24 nt small RNA class is more diverse (has more unique reads) and has less redundancy (lower counts for each unique reads) than the 21 nt small RNA class. A total of 1,649,996 unique reads were then submitted to the downstream small RNA analysis pipeline outlined in Fig. 1, step 4 onwards. Although *P. aphrodite* genomic data is unavailable in public databases, over 230,000 *P. aphrodite* transcripts in our Orchidstra database (Su et al. 2011) were able to provide reference sequences for mapping of small RNA reads. In total, 220,893 unique reads were mapped to Orchidstra transcripts among which 53,096 and 167,797 unique reads mapped to protein-coding ESTs and unknown EST/non-coding transcripts, respectively. The unique reads that mapped to protein-coding ESTs are probably highly degraded mRNA fragments or siRNA. The unique reads that did not match protein-coding ESTs were BLAST searched against the Rfam database (Gardner et al. 2011). Approximately 3.9 % of 1.65 million unique reads (63,602 reads) were tRNA, rRNA, small nuclear RNA (snRNA), small nucleolar RNA (snoRNA) or other small RNA molecules (not miRNA). In order to identify conserved miRNAs, the remaining reads and the reads that mapped to unknown ESTs/non-coding transcripts were searched against the plant miRNAs downloaded from the microRNA database (miRBase, http://www.mirbase.org) (Kozomara and Griffiths-Jones 2011). A total of 3,251 unique reads showing less than three mismatches with a known plant miRNA were identified, among which 447 unique reads were perfectly aligned with known miRNAs. Furthermore, we identified 23 new miRNAs by using the approach described in the Materials and methods section. Table 2 shows the annotation and classification of unique reads and the abundance of different small RNA categories in the sequenced small RNA libraries.

**Table 1:** Summary of small RNA next generation sequencing data processing

Processing steps	Root	Leaf	Flower	Seed	Total
*Input*					
Unique reads	6,755,739	6,901,052	8,538,019	5,798,159	22,829,317
Total reads	23,852,494	24,059,282	23,741,532	25,494,472	97,147,780
*After removing adaptors and poly-A/T/C/G/N*					
Unique reads	5,787,223	5,802,483	7,347,504	4,856,499	19,207,362
Total reads	20,618,934	20,514,477	20,496,815	22,312,078	83,942,304
*After filtering out reads with low counts*					
Unique reads	1,193,873	1,161,516	1,231,736	1,079,437	1,697,668
Total reads	15,463,951	15,293,812	13,668,993	18,097,032	62,523,788
*After removal of virus/chloroplast sequences (reads entered into the annotation pipeline)*					
Unique reads	1,164,475	1,117,720	1,206,840	1,047,951	1,649,996
Total reads	15,117,306	13,507,134	13,408,510	17,354,424	59,387,374

**Table 2:** Distribution of unique reads in the sequenced *P. aphrodite* small RNA libraries

Libraries/small RNA	Root		Leaf		Flower		Seed	
	Total	Unique	Total	Unique	Total	Unique	Total	Unique
Protein-coding EST	989,288	38,231	861,761	36,576	799,267	39,707	840,605	38,459
Non-coding EST	4,692,205	131,039	4,401,161	122,760	3,862,859	133,855	4,228,824	120,271
rRNA	916,073	26,320	584,814	23,521	627,592	23,344	1,066,465	26,117
tRNA	1,531,792	15,873	858,608	13,740	1,851,199	16,072	4,577,493	19,705
snRNA	7,116	987	6,963	830	24,549	1,199	111,365	1,678
snoRNA	13,180	1,148	6,948	961	9,133	1,128	27,606	1,399
miRNA	1,583,670	2,376	3,506,031	2,661	1,703,039	2,335	2,567,995	2,466
other sRNAs	73,124	2,897	50,041	2,750	56,033	2,759	115,409	3,117
Unmatched	6,403,763	945,604	5,318,140	913,921	5,901,112	986,441	5,032,560	834,739

### Transcriptome-wide identification of microRNAs in *P. aphrodite*

Alignment of unique small RNA reads with experimentally verified plant miRNAs resulted in a total of 3,251 unique reads for 181 known miRNAs. These orchid miRNAs were classified into 88 known plant miRNA families (Supplemental Figure 3). To date, 33 of these miRNA families have been identified in at least three green plant species in the miRBase (based on the data downloaded from miRBase). The *P. aphrodite* miRNAs showed high sequence similarity to their homologs in *Zea mays*, *Oryza sativa*, *A. thaliana*, and *Vitis vinifera*. Many highly conserved miRNA families were identified in the four libraries studied, such as miR156/157, 159/319, 165/166, 170/171, 160, 168, 172, 396, and 399.

Fifty-two known miRNA families found in this study overlapped with those found in a previous study of miRNA in *P. orchid* (An et al. 2011), despite the tissues, conditions, annotation criteria and read-count filtering method used in our current study being quite different from the earlier investigation. This study further identified additional 36 known miRNA families (Supplemental Figure 3) that were not found in the previous study (An et al. 2011). These miRNA families may correspond to the tissues and developmental stages analysed. In addition, miRNA with low expression levels were detected by deep sequencing in this study. Among the 36 miRNA families identified in our data but not in (An et al. 2011), miR3440 are conserved between *P. aphrodite*, *Arabidopsis lyrata*, *A. thaliana* and *Helianthus annuus*. Moreover, miR774, miR4221, miR5654 and miR2950 are conserved between *P. aphrodite* and 2 other plant species in the miRBase (see Supplemental Figure 3 for details of the plant species distribution in the miRBase for each miR family). These results indicate that these miRNA families have homologs in both eudicot and monocot species. Furthermore, 6 miRNA families (miR2868, 2905, 2931, 5155, 5532 and 5538) are conserved between *P. aphrodite* and *O. sativa* but not reported in eudicot species.

Known orchid miRNAs accounted for 10.5, 26.0, 12.7, and 14.8 % of the total small RNAs (after all filtering steps) in root, leaf, flower and seed, respectively. One of the most conserved miRNAs, miR159, is known to play roles in plant development and fertility (Jones-Rhoades et al. 2006). It has been reported that miR159 accumulates in *P. aphrodite* stalks (An et al. 2011). Of the four tissues investigated, the miR159 family showed the highest expression levels of all the miRNA families across all tissue types, the occurrence of miR159 varied from 114,828 RPM in leaf to 31,884 RPM in seed (Table 3). When the abundance of a single miRNA family was calculated as a percentage of the total number of miRNAs in each tissue, the miR159 family alone accounted for 38.6, 44.2, 66.0 and 21.6 % of the total miRNA in the root, leaf, flower and seed libraries, respectively. Several other miRNA families, such as miR528 and miR535 families, also had high abundance of expression across all tissue samples, and the miR156 family was highly expressed in the seed library. Closer inspection revealed tissue-specific differential expression among individual miRNAs within the miR156 family. The majority of miR156 reads (about 95 %) in seeds was a perfect match to miR156a, while miR156b was enriched (tenfold more abundant) in the roots relative to seeds.

**Table 3:** The top 30 miRNA families expressed at highest levels in *P. aphrodite*

Family/library	miRNA expression levels (reads per million, RPM)			
	Root	Leaf	Flower	Seed
miR159	40,458.60	114,828.36	83,771.72	31,884.38
miR528	3,583.11	87,059.25	15,639.47	70,731.99
miR535	47,988.38	41,264.12	14,482.89	7,607.86
miR156	2,340.83	595.61	81.66	32,722.32*
miR166	1,788.48	1,918.84	3,940.71	1,489.36
miR162	2,409.82	3,217.41	1,521.87	423.24
miR171	29.11	1,872.34	3,181.79	651.13
miR167	1,683.43	3,355.12	124.03	190.67
miR319	991.71	366.55	1,967.85	1,280.60
miR396	612.74	2,577.08*	235.45	108.16
miR894	1,474.60	891.75	404.37	234.64
miR164	678.36	219.74	535.33	45.58
miR408	39.56	619.38	285.27	227.61
miR168	273.53	215.00	211.21	66.78
miR5139	219.22	232.77	151.62	125.79
miR529	48.88	8.00	80.77	54.74
miR172	1.72	46.86	147.52*	1.15
miR397	4.43	69.52	28.79	46.73
miR2950	47.16	50.86	49.07	1.04
miR394	5.82	10.59	25.73	14.87
miR398	1.19	38.72*	6.79	2.02
miR160	5.95	14.51	22.45	2.19
miR165	9.59	7.26	12.31	1.61
miR858	0.33	0.07	24.09*	0.12
miR783	6.48	8.96	4.03	0.63
miR2911	8.14*	0.52	1.34	2.25
miR1318	1.98	2.74	7.91	0.46
miR395	0.13	1.92	9.99*	0.58
miR399	0.99	5.55	0.60	2.48
miR3946	5.16	2.37	0.15	0.69

The closer the SPM is to 1 the greater the extent of tissue specificity

* Specificity measure (SPM) >0.9

The sequencing reads of the three most abundant miRNAs (miR535, miR159, and miR528 in roots, leaves, and flowers; and miR528, miR156, miR159 in seeds) constituted over 87 % of the total known miRNA reads in all tissue types, indicating that they are likely ubiquitous in *P. aphrodite*. The miR162, miR167, miR396, miR845, and miR894 families had higher expression levels in root and leaf libraries, while miR319 and miR529 families had higher expression in flower and seed libraries.

### Tissue-specific expression of orchid miRNAs

**Fig. 3:**
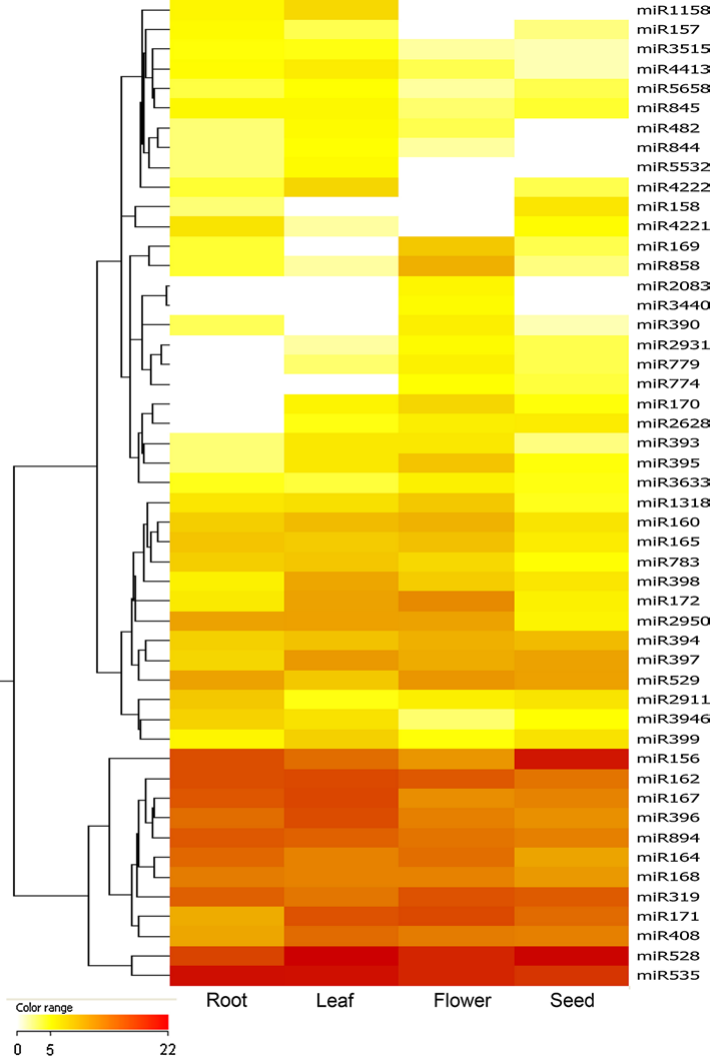
Heat map and cluster dendrogram of 50 differentially expressed miRNAs. The heat map summarizes the expression of 50 differentially expressed miRNAs across *P. aphrodite* tissues. Clustering was based on Euclidean distance and centroid linkage rule. miRNAs exhibiting a fold change of at least 4.0 were selected for cluster analysis

Since deep sequencing produced a large number of reads, the read abundance in the libraries could be used to perform miRNA digital gene expression profiling (DGE) in *P. aphrodite*. Fifty miRNAs that exhibited fold-change of at least 4.0 were subjected to cluster analysis to show their expression patterns (Fig. 3). In order to reveal patterns of specific enrichment of miRNAs in different tissue types, SPM, a quantitative estimate of the tissue specificity of a gene in a profile (Xiao et al. 2010), was calculated for each miRNA family in each tissue. SPM score ranges from 0 to 1, the closer the SPM score is to 1, the higher the tissue specificity. Twenty-eight out of 88 miRNA families had an SPM above 0.9 in one of the four tissues. Among the tissue-specific expression miRNAs (with SPM >0.9), miR398 and miR396 were found primarily in leaves while miR2911 was over-expressed in roots. miR172, miR169, miR858 and miR395 were found to be expressed predominantly in flowers. The miR156 family was overexpressed in seeds (32,722 RPM) and under-expressed in flowers (82 RPM). In contrast, miR172 levels were higher in flowers (148 RPM) than in leaves (47 RPM), and were expressed at very low levels in roots and seeds (Table 3). miR156 is known to promote juvenile development by repressing members of the SQUAMOSA promoter-binding-like (SPL) family of transcription factors (Xie et al. 2006). In Arabidopsis levels of expression of miR156 are highest in seedlings and decline during development. In contrast, expression of miR172 is low in young seedlings and gradually increases throughout the life cycle, showing an opposite expression pattern to miR156 (Wu et al. 2009) [and reviewed in Huijser and Schmid (2011)]. The results of our study indicate that miR156 and miR172 are also expressed in inverse patterns in orchid, consistent with the findings in Arabidopsis (Wu et al. 2009) and maize (Chuck et al. 2007). The targets of miR156 and miR172 were identified through our target prediction procedure.

### Identification of *P. aphrodite* miRNA precursors

74 putative miRNA precursors were predicted for 38 miRNA families. 73 out of our 74 precursors, except the precursor PATC130914 of miR396, were not reported in the previous study (An et al. 2011) which predicted 14 putative precursors. The lengths of out predicted pre-miRNA hairpin structures varied from 55 to 293 nt with an average length of 123 nt. The MFEs of their hairpin structures range from −12.72 to −198.14 kcal/mol with an average value of −58.65 kcal/mol. The average MFE of *P. aphrodite* was similar to the MFE of Arabidopsis precursors. Twenty-two miRNA-star sequences (miRNA*), the complementary strands of functional mature miRNA, were also detected in our libraries. The predicted miRNA precursors, their families and the corresponding sRNA reads are shown in Table 4. Supplemental Figure 4 illustrates the alignments of orchid miR166 with its homologs in other species (Supplemental Figure 4a and 4c), and the stem-loop structure of the predicted precursor of miR166 (Orchidstra ID: PATC143861) as an example (Supplemental Figure 4b).

**Table 4:** Potential miRNA precursors of conserved miRNA in *P. aphrodite*

miR family	Precursor ID	Length	Position	MFE (Kcal/mol)	miRNA source	miR family	Precursor ID	Length	Position	MFE (Kcal/mol)	miRNA source
miR156	PATC127202	110	59–168	−66.56	P^a^	miR1533	PATC082198	130	41–170	−35	M
miR156	PATC133131	107	217–323	−57.13	P	miR1533	PATC082683	223	1–223	−132.48	M
miR156	PATC135255	107	217–323	−57.13	P	miR-24	PATC178264	125	128–4	−108.57	M
miR156	PATC147904	108	1,193–1,086	−67.86	P	miR-2304	PATC106586	82	86–167	−34.34	M
miR156	PATC164883	107	320–214	−65.99	P	miR-2345	PATC034115	69	253–185	−23.4	M
miR158	PATC050600	219	198–416	−58.22	M^b^	miR-2359	PATC166661	114	286–173	−56.45	M
miR159	PATC154123	242	321–80	−105.04	P	miR2361	PATC130801	217	1,659–1,443	−70.66	M
miR160	PATC024352	104	72–175	−52.08	P	miR-2445	PATC093130	125	144–268	−41.74	M
miR160	PATC035616	100	112–13	−50.23	P	miR390	PATC042111	239	239–1	−109.49	M
miR160	PATC069443	106	143–38	−57.97	P	miR394	PATC230021	100	91–190	−44.42	P
miR162	PATC154721	136	1,791–1,656	−64.78	P	miR394	PATC230433	100	94–193	−44.42	P
miR166	PATC143861	140	3,037–2,898	−63.8	P	miR396	PATC130914	101	33–133	−56.23	P
miR167	PATC043563	90	150–61	−45.47	P	miR399	PATC158376	225	1,084–860	−122.48	P
miR167	PATC151450	293	702–410	−109.64	P	miR-3,141	PATC075139	144	188–45	−123.39	M
miR168	PATC138003	96	100–195	−71.34	P	miR-466	PATC091142	135	409–275	−44.66	M
miR168	PATC161629	107	3–109	−61.87	P	miR-466	PATC103125	66	95–30	−41.86	M
miR168	PATC193994	75	47–121	−44.59	P	miR-466	PATC118951	68	420–353	−27.53	M
miR169	PATC114606	108	419–312	−45.4	M	miR-466	PATC120777	55	1–55	−27.43	M
miR169	PATC194844	109	45–153	−54.42	P	miR-466	PATC144413	136	174–39	−35.63	M
miR169	PATC195106	110	72–181	−54.33	P	miR-466	PATC186790	72	140–211	−13.5	M
miR171	PATC106872	118	146–29	−49	P	miR-466	PATC192812	107	37–143	−34.51	M
miR171	PATC151283	99	340–438	−50.89	P	miR-466	PATC155125	74	264–191	−48.23	M
miR171	PATC152062	105	468–364	−49.56	P	miR-466	PATC158916	80	145–66	−34.52	M
miR172	PATC181476	115	149–35	−53.38	P	miR-466	PATC183646	225	1–225	−79.49	M
miR172	PATC101490	86	102–187	−44.61	P	miR-466	PATC226024	90	173–84	−38.95	M
miR-1187	PATC134846	209	66–274	−72.48	M	miR-467	PATC095237	187	203–17	−118.01	M
miR-1187	PATC155430	82	150–231	−30.72	M	miR-4044	PATC035708	96	339–244	−75.66	M
miR-1187	PATC155746	89	407–495	−36.33	M	miR4077	PATC212682	91	39–129	−26.03	M
miR-1187	PATC159725	216	389–174	−62.96	M	miR-4307	PATC113165	78	145–68	−22.02	M
miR-1187	PATC160392	144	349–206	−42.85	M	miR4382	PATC172094	71	150–220	−18.12	M
miR-1187	PATC160599	124	119–242	−53.8	M	miR528	PATC153398	103	13–115	−52.08	P
miR-1187	PATC177152	105	105–1	−36.62	M	miR529	PATC150157	101	1,265–1,165	−43.22	M
miR-1187	PATC217328	87	180–266	−27.25	M	miR535	PATC151754	101	672–772	−54.3	P
miR-1187	PATC218556	95	103–9	−33.69	M	miR535	PATC196028	106	21–126	−62.5	P
miR-1281	PATC081629	204	1–204	−198.14	M	miR-669	PATC082299	94	132–225	−39.8	M
miR1432	PATC023631	89	146–58	−47.02	P	miR-669	PATC230197	70	270–201	−12.72	M
miR1530	PATC178976	119	169–51	−27.78	M	miR845	PATC103597	164	71–234	−44.37	M

^a^Prediction based on the *P. aphrodite* annotated miRNA data of the current study

^b^Prediction based on publicly known miRNA sequences in the miRBase

### Identification of microRNA targets in *P.aphrodite*

We used two approaches to identify miRNA targets in *P. aphrodite*. The first approach (see Target prediction procedure section) predicts 160 miRNA target transcripts for 35 known miRNA families in *P. aphrodite* without relying on known targets of other organisms. Many miRNAs found in Arabidopsis have potential homologs in rice and other monocotyledonous plants (Reinhart et al. 2002), and computational analyses have predicted that these miRNAs might regulate homologous targets in several species (Jones-Rhoades and Bartel 2004; Sunkar et al. 2005; Bonnet et al. 2004; Adai et al. 2005; Wang et al. 2004). Based on the premise that miRNA targets are conserved across different plant species, another approach for target identification is to search for homologs among known target genes. Using this second approach we identified 95 target transcripts for 28 known miRNA families appearing in our small RNA libraries. Processing through our computational pipeline resulted in a total of 228 predicted target genes from 46 miRNA families. Of the predicted miRNA targets, 27 target genes from 12 conserved miRNA families were identified by both methods.

These two approaches led to somewhat different results due to the following reasons. First, protein-coding ESTs are often non-full-length/truncated and the miRNA-binding site related sequences are missing in the existing database. This limits the target prediction in the first approach because it relies on the direct alignment of miRNAs to EST sequences, while the second approach relies on target homology at the amino acid level and thus the detection sensitivity is less affected by non-full-length sequences. Second, the second approach searched only the homologs of those previously reported targets of known miRNAs. There were 18 miRNA families that have 72 targets being detected by the first approach while the second approach could not be applied to these miRNAs because they were not reported in Arabidopsis or their targets were not found in ASRP database. Third, the second approach cannot find orchid specific targets. Given that each of the approaches has its own strengths and limitations, using both homology-dependent and homology-independent approaches resulted in more comprehensive target identification than using either one alone, especially for non-model organism without complete transcriptome sequence data. Supplemental Figure 2b illustrates an example of a target site that is conserved in *P. aphrodite* and Arabidopsis. 196 out of our 228 predicted targets were not reported in the previous study (An et al. 2011), the other 32 predicted targets share same Arabidopsis homologs with the targets reported in (An et al. 2011). Table 5 shows a list of miRNA targets identified in this study and their targets reported in Arabidopsis.

**Table 5:** Predicted gene targets of miRNA in *P. aphrodite*

miRNA family	Targets predicted by method 1	Targets predicted by method 2	Targets in Arabidopsis
miR156/157	PATC133938,PATC012624,PATC136943,PATC024123,PATC042847,PATC149565,PATC130083,PATC144388,PATC147320,PATC134878,PATC141616,PATC161070,PATC135103,PATC049704,PATC136866,PATC135707*,PATC153064*,PATC143407*,PATC148826*,PATC141751*	PATC231283,PATC232246,PATC203938,PATC229081,PATC136640,PATC133464,PATC198877,PATC135707*,PATC153064*,PATC143407*,PATC141751*,PATC148826*	AT5G50570.2,AT3G15270.1,AT5G43270.1,AT5G43270.3,AT2G42200.1,AT5G50670.1
miR158	N/A	PATC029643,PATC022855	AT3G03580.1
miR159	PATC139252,PATC127723,PATC156552,PATC146639,PATC143380,PATC128406,PATC131148,PATC128474,PATC089381,PATC145355,PATC007115,PATC155837,PATC023828,PATC224753,PATC148783*	PATC128977,PATC137824,PATC072039,PATC148783*	AT3G11440.1,AT2G32460.1,AT5G06100.3,AT5G55020.1
miR160	PATC088662,PATC134082*,PATC136630*	PATC130364,PATC134082*,PATC136630*	AT4G30080.1
miR162	PATC147200,PATC135020	PATC140870	AT1G01040.1
miR164	PATC132261,PATC133411, PATC088390,PATC152024*,PATC132958*,PATC155244*	PATC142438,PATC124714,PATC152024*,PATC132958*,PATC155244*	AT3G12977.1,AT5G61430.1,AT1G56010.2
miR165/166	PATC115701,PATC152414*,PATC129561*,PATC144912*,PATC146998*	PATC123294,PATC129561*,PATC152414*,PATC144912*,PATC146998*	AT2G34710.1,AT4G32880.1
miR167	PATC137831,PATC142408,PATC139566,PATC141313,PATC022536	PATC134326,PATC140885	AT1G30330.2,AT1G30330.1
miR168	PATC143303*	PATC157237,PATC157394,PATC202515,PATC093469,PATC213155,PATC155439,PATC129162,PATC143303*	AT1G48410.2,AT1G48410.1,AT1G48410.3
miR169	N/A	PATC129283,PATC126444,PATC150676	AT5G06510.2,AT1G54160.1
miR170/171	PATC049290,PATC154363,PATC023657,PATC124992,PATC143284*,PATC149190*,PATC149532*	PATC144573,PATC130737,PATC143284*,PATC149190*,PATC149532*	AT4G00150.1
miR172	PATC148983,PATC233147,PATC133311*,PATC135984*,PATC124448*	PATC147704,PATC133311*,PATC135984*,PATC124448*	AT4G36920.2,AT2G28550.3,AT4G36920.1,AT2G28550.2
miR319	PATC139252,PATC125994,PATC148783,PATC007115,PATC129076,PATC023828,PATC131148,PATC128474,PATC089381	PATC129038,PATC135086,PATC133065,PATC134871,PATC136074,PATC138402,PATC038597	AT1G30210.1,AT3G15030.3,AT4G18390.2,AT1G30210.2,AT1G53230.1
miR393	PATC157696*,PATC149677*	PATC157696*,PATC149677*	AT3G62980.1
miR394	PATC129841*	PATC129841*	AT1G27340.1
miR395	PATC147276*	PATC126179,PATC147276*	AT4G14680.1
miR396	PATC131246,PATC125749,PATC125758,PATC129482,PATC152055,PATC150890,PATC129416,PATC151850,PATC149114,PATC127048,PATC137791,PATC149117,PATC130150,PATC076655,PATC144370	PATC130257,PATC144187,PATC150161,PATC133318	AT4G37740.1,AT2G22840.1,AT2G36400.1
miR397	N/A	PATC145076,PATC125841,PATC132212,PATC124360,PATC130366,PATC140962	AT5G60020.1,AT2G38080.1,AT2G29130.1
miR398	N/A	PATC143343,PATC152430,PATC143977	AT3G15640.2,AT2G28190.1
miR399	PATC145005,PATC143740*	PATC143740*	AT2G33770.1
miR408	PATC128673,PATC149690	PATC113957,PATC146846,PATC003794	AT2G02850.1,AT2G30210.1
miR528	PATC156396,PATC127027	N/A	N/A
miR529	PATC143407,PATC134878,PATC135103,PATC153064,PATC148826	N/A	N/A
miR535	PATC128688,PATC135523,PATC138531,PATC152377,PATC134561,PATC130349,PATC129893,PATC154063,PATC151754,PATC127615,PATC138376,PATC127350,PATC157118,PATC142441,PATC128040,PATC126341,PATC138729,PATC147167,PATC133246,PATC145620,PATC130662,PATC146667	N/A	N/A
miR774	PATC126979	N/A	N/A
miR779	N/A	PATC000784,PATC172415,PATC137533,PATC024708,PATC025304,PATC167710,PATC128352	AT5G53890.1
miR783	PATC205714,PATC125598	N/A	N/A
miR827	N/A	PATC132672	AT1G02860.1
miR844	N/A	PATC208749	AT5G51270.1
miR845	PATC132451	N/A	N/A
miR858	N/A	PATC041804,PATC124665,PATC129048	AT2G47460.1,AT3G08500.1
miR1061	PATC152227	N/A	N/A
miR1318	PATC124801,PATC148138	N/A	N/A
miR2628	PATC145891	N/A	N/A
miR2950	PATC127350	N/A	N/A
miR4384	PATC141931	N/A	N/A
miR4413	PATC038751	N/A	N/A
miR5021	PATC140350,PATC124700,PATC125225,PATC151681,PATC130999,PATC124610,PATC150529,PATC069316,PATC155090,PATC126852,PATC127170,PATC150919,PATC140968,PATC137680,PATC155795,PATC092129,PATC152249,PATC125628,PATC123011	N/A	N/A
miR5139	PATC143291,PATC156350,PATC157217,PATC139307,PATC022793,PATC158299,PATC236030	N/A	N/A
miR5204	PATC126586	N/A	N/A
miR5253	PATC138734,PATC048488	N/A	N/A
miR5658	PATC139976,PATC124238	N/A	N/A
miR5661	PATC028326	N/A	N/A

Some miRNAs have detectable sequence similarity and share common predicted targets. For example, miR156 and miR529 have overlapping predicted target sites in PATC134878 and PATC135103, both of which belong to SPL family of transcription factors. Our prediction results are consistent with previous reports showing that both miR156 and miR529 have similar predicted targets consisting mainly of SPL genes in maize (Zhang et al. 2009b), and analysis showing the cleavage of tsh4 (encoding a SBP-box transcription factor) transcript by miR529 and miR156 (Chuck et al. 2010). Another example of overlapping targets comes from miR159 and miR319, which share high sequence similarity. These two miRNAs have similar processing mechanisms (Bologna et al. 2009) and may have evolved from a common ancestor (Li et al. 2011). Our prediction showed that miR159 targets transcripts from the MYB family while miR319 targets the TCP transcription factor family. These results agree with findings in other organisms (Palatnik et al. 2007). In addition, miR159 and miR319 have five overlapping predicted targets, including two MYB genes (Achard et al. 2004) and three kinase genes involved in post-translational modification (de la Fuente van Bentem et al. 2008). It has been reported that MYB genes are occasionally targeted by miR319a in Arabidopsis wild-type plants, but due to low expression of miR319 and much higher expression levels of miR159, MYBs are predominantly targeted by miR159 (Palatnik et al. 2007). The overlapping targets obtained from our prediction are consistent with observations in Arabidopsis and suggest that the expression of some mRNAs maybe regulated by coordinated actions of multiple miRNAs in *P. aphrodite*. Compared with other genomes, data available for *P. aphrodite* is relatively limited, which restricts the search for miRNA genes and their targets. Our bioinformatics analysis using the deep sequencing data and EST data revealed many target genes and miRNA precursors not previously reported.

### New miRNAs and their target genes

To identify novel miRNAs, unique sRNA reads were mapped to unknown/non-coding EST in our Orchidstra database. Only perfect matches were allowed. The secondary structures of the matched ESTs were predicted by Mfold. The filtering process was similar to that used in the precursor prediction of known miRNAs, with an additional filter added. After hairpin structure analysis, following the recommendations of the previous study (Meyers et al. 2008), the complementary strands of potential mature miRNAs were searched against our small RNA libraries to find the miRNA-star counterparts. The results from previous studies of miRNA annotation confidence in human NGS datasets also supported the notion of Meyer’s criteria (Hansen et al. 2011). Our concern is not solely with the false negative rate but also with the false positive rate, especially millions of hairpin structures can be found in a large eukaryotic genome. This miRNA star filter greatly reduced the number of candidate miRNA stem-loop structures. We obtained 23 new miRNA candidates from 17 predicted miRNA precursors and identified their miRNA* in our small RNA libraries. The precursor lengths of the new miRNAs ranged from 74 to 228 nt, with an average length of 128 nt (Table 6). All the predicted precursors could fold into a characteristic stem-loop structure with the mature miRNA on either the 5′ arm or the 3′ arm of the precursor (Supplemental Figure 5). The size of the predicted mature miRNAs ranged from 20 to 24 nt. We inspected the predicted new mature miRNA sequences and found that 19 out of the 23 miRNAs started with a uridine (U). U at the 5′ end is a feature shared by most known miRNAs. These new miRNAs were not found in other plant species in the miRBase v.18 that we used to perform miRNA annotation. However, the miRNA PA-miR1-5p has a rice homolog that was identified as a new miRNA in a recent study (Jeong et al. 2011) but not presented in miRBase v.18. Homologs of these new miRNAs were further identified by using precursors as queries to BLASTN against the genomic sequences and EST database of NCBI with a cutoff value of 1e-4. We found homologs of PA-miR2 in *Dendrobium nobile*, *O. sativa*, *Oryza longistaminata*, and *Solanum tuberosum*. Some homologs of new miRNAs were only found in orchid species, including homologs of PA-miR10 in *Phalaenopsis violacea*, and homologs of PA-miR12 and PA-miR13 in *Phalaenopsis equestris*. All homologs in other plants contained the sequences that are identical or with one mismatch to their mature miRNA counterparts in *P. aphrodite*, and 85–97 % alignment identities over at least 50 % of the precursor length.

**Table 6:** *P. aphrodite* novel miRNAs identified in this study and their target genes

miR ID	PASR ID	Size	Mature microRNA sequence	Precursor source	Precursor length (nt)	MFE (Kcal/mol)	Target
PA-miR1-5p	PASR10280190	21	UUUUGCUCAAGACCGCGCAAC	PATC206486	194	−83.52	PATC133864
							PATC138350
							PATC154853
PA-miR1-3p	PASR18220033	21	UGCGUGGUCUUGCGCAAGAUA	PATC206486	194	−83.52	
PA-miR2-5p	PASR04371124	20	UGCAUAGCUUGUAAGAAGCC	PATC142657	105	−60.07	
PA-miR2-3p	PASR17739734	21	UUUUCUUGCAAGUUAUGCAGC	PATC142657	105	−60.07	PATC131704
							PATC129763
PA-miR3-5p	PASR11327008	21	UGCAUCAGUACUCCAAUGAGG	PATC207842	111	−92.14	PATC145358
							PATC150760
PA-miR3-3p	PASR06328657	21	UCAUUGGAGUACUGUUGCAUC	PATC207842	111	−92.14	
PA-miR4-5p	PASR18109326	24	UUCCAUGUAGUAGUCUUGACCGCU	PATC138134	76	−35.09	
PA-miR5-5p	PASR07098857	22	UUUUACCUAAAAAUAGAACAGG	PATC121951	174	−58.56	
PA-miR5-3p	PASR10145605	22	UGUUUUGUUCUUGGUUACUAGU	PATC121951	174	−58.56	PATC068360
PA-miR6-3p	PASR00230146	22	UAAUUUGGAUGAUACAAAGAGC	PATC024195	119	−68.46	
PA-miR7-3p	PASR10868151	24	AGAUCACUGUGAUCUACAGUCAGG	PATC149891	141	−60.19	PATC135091
							PATC153366
PA-miR8-5p	PASR04318366	21	UGAUUUUGGGGUACUAUAUGA	PATC197748	74	−48.22	
PA-miR9-5p	PASR12483558	24	AUUGUCCGGACAAUAGAUGCGAUA	PATC057734	115	−63.01	
PA-miR9-3p	PASR01446209	24	UUGCAUCUAUUGUCUGGACACUGG	PATC057734	115	−63.01	
PA-miR10-5p	PASR07436675	24	UAAGUUAACGAGCCGAACACGAAC	PATC222425	85	−48.81	
PA-miR11-3p	PASR12661840	22	UCAGGGGUACAAUUGUUUCUAG	PATC078804	178	−107.96	
PA-miR12-5p	PASR03044487	22	AUUUCAAUGAUGGGAUGCCAUG	PATC151638	114	−53.53	
PA-miR12-3p	PASR09253774	22	UGGCGUUCCAUCAUGGAAGUCC	PATC151638	114	−53.53	
PA-miR13a-5p	PASR16899997	21	UCGUGUUCGGUUCGUCAAUGA	PATC223607	108	−66.61	
PA-miR13b-5p	PASR16899997	21	UCGUGUUCGGUUCGUCAAUGA	PATC211404	109	−72.32	
PA-miR14-5p	PASR18104423	24	UAGAUAACGAUUGAGAGAUUAGUG	PATC225301	85	−19.36	
PA-miR14-3p	PASR16127551	22	UUAAGCUUUGAAUCAAUUUAAA	PATC225301	85	−19.36	PATC174550
PA-miR15-3p	PASR16174279	24	AAAUCCCAUGUUUAAGCUCACAUC	PATC100629	159	−71.46	
PA-miR16-5p	PASR03747890	21	UCCUUACUCUCCAAAAAUGGG	PATC154123	228	−103.76	PATC135587

Several new miRNA exhibited different tissue specificities with SPM >0.9. For example, PA-miR2 (8.24 RPM in seed), PA-miR6 (1.33 RPM in leaf) and PA-miR15 (0.46 RPM in root) were specific to seed, leaf, and root, respectively. PA-miR1 (1.34 RPM in flower), PA-miR3-3p (0.89 RPM), PA-miR7 (0.97 RPM), and PA-miR16 (0.45 RPM) were specifically expressed in the flower. A total of 12 targets were predicted for 7 new miRNAs. Three PA-miR1 targets (PATC133864, PATC138350, and PATC154853) code for DEFICIENS-like MADS-box transcription factors that are involved in flower development and patterning (reviewed in (Krizek and Fletcher 2005)). The targets of PA-miR2 are MIKC-type MADS-box transcription factor (wheat WM30 homolog) genes. Other targets of new miRNAs include proteins that function in signal transduction, members of the transferase families, transporters, and transposable element-related proteins.

The detailed information of new miRNAs (and conserved miRNAs) has been incorporated into the Orchidstra database and is available via web access (Supplemental Figure 6). For each miRNA, Orchidstra database contains the following information: sequence information (Supplemental Figure 6a), expression levels (Supplemental Figure 6b), miRNA targets (Supplemental Figure 6c), stem-loop structure of miRNA presursors (Supplemental Figure 6d, e), and visualization of deep sequencing reads that were mapped to the miRNA presursors (Supplemental Figure 6f). Orchidstra provides internal links between data resources, to precursors and to targets with more detailed information.

### Experimental validation of miRNA-directed cleavage sites within target transcripts

**Fig. 4:**
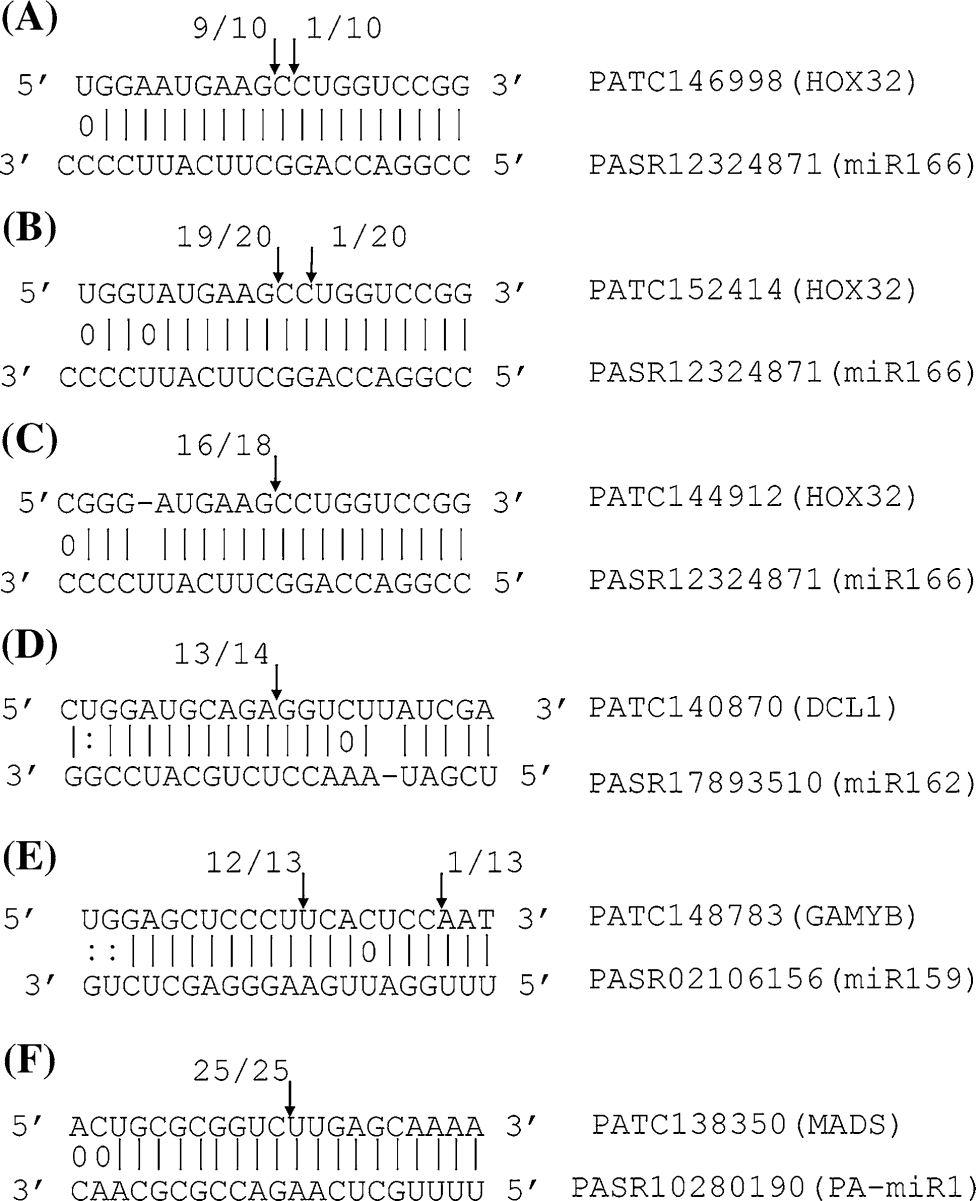
Validation of miRNA target genes in *P. aphrodite* using 5′ RACE and sequencing. miRNA-binding sites in target genes are aligned with the corresponding miRNAs. *Arrows* indicate the cleavage sites determined by sequencing of 5′ RACE clones, and the numbers indicate the fraction of cloned 5′ RACE products corresponding to each site. Canonical pairings are indicated by *solid lines*. G–U pairings and non-canonical pairings are indicated by *colons* and *circles*, respectively. **a** miR166 and its target PATC146998, showing positions 1061–1080 on the EST. **b** miR166 and its target PATC152414, showing positions 312–331 on the EST. **c** miR166 and its target PATC144912, showing positions 348–367 on the EST. **d** miR162 and its target PATC140870, showing positions 3358–3379 on the EST. **e** miR159 and its target PATC148783, showing positions 1062–1082 on the EST. **f** miR319 and its target PATC148783, showing positions 1061–1081 on the EST. **g** PA-miR1 and its target PATC138350, showing positions 372–392 on the EST

RLM-5′ RACE experiment was performed on a subset of target genes to validate miRNA-directed cleavage sites within the target transcripts (Fig. 4). PATC146998, PATC152414 and PATC144912, homologs of rice homeobox-leucine zipper protein HOX32, were identified as the targets of miR166 (Fig. 4a–c). MiR162-mediated cleavage in PATC140870 (Dicer-like protein 1, DCL1), miR159-mediated cleavage in PATC148783 (homolog of GAMYB transcription factor), and PA-miR1-mediated cleavage in PATC138350 (DEFICIENS-like MADS-box transcription factor) were also detected in the RLM-5′ RACE procedure (Fig. 4d–f). Sequence analysis of the 5′ RACE cleaved product showed that the cleavage in orchid targets mainly occurred at the site opposite the 10th and 11th nucleotides from the 5′ end of miRNA. The predicted targets of both known and new miRNAs were validated by RLM-5′ RACE, indicating that our pipeline provided the sensitivity to detect miRNAs and their targets in orchid.

### Functional classification of miRNA targets in *P. aphrodite*

**Fig. 5:**
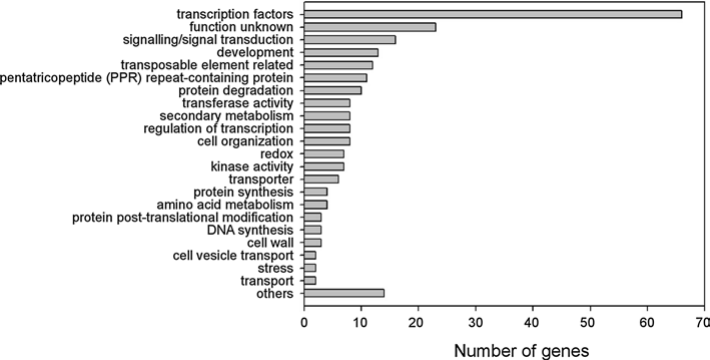
MapMan functional category classification of miRNA-target genes in *P. aphrodite*

Our target prediction procedure predicted 240 miRNA targets in total (228 targets for known miRNAs and 12 targets for newly identified miRNAs). The target list contained many previously identified targets, such as the SPL genes as targets of miR156, the MYB genes for miR159, auxin response factors for miR167, the AP2 genes for miR172, and the PHO2 genes for miR399. New targets were also identified, such as tubulin-specific chaperone for miR528, oxidoreductase gene for miR399, and MADS-box transcription factors for new miRNA PA-miR1 and PA-miR2. Functional classification of the predicted miRNA targets was conducted by combining the results from the Gene Ontology (GO, http://www.geneontology.org/), Pfam (http://pfam.sanger.ac.uk) searches, and MapMan (http://mapman.gabipd.org/web/guest/home) ontology annotation transferred from Arabidopsis homologs. One hundred and ninety-four of the 240 targets contained at least one pfam domain, and 212 targets were assigned at least one GO or MapMan ontology. For each target the functional annotation and the assignment of GO terms can be found in the target pages in the Orchidstra database. Of the predicted target genes, 27.5 % are members of transcription factor families, 9.5 % encode proteins of unknown function or are proteins of unknown biological process, 6.7 % encode proteins involved in signalling or signal transduction and 5.4 % are development-related genes (Fig. 5). The other predicted target genes fell into a further 19 functional categories that include many diverse functions and biological processes such as protein degradation, secondary metabolism, redox, transferase, kinase, transporter and transposable element, suggesting that miRNAs regulate a wide range of biological activities in *P. aphrodite*. Further inspection found that target genes were significantly enriched (*P* < 2.2e-16; Fisher’s Exact Test) in transcription factors relative to the proportion of transcription factors among all *P. aphrodite* protein-coding genes. The signalling/signal transduction term is also over-represented (*P* < 8.8e-13). Meanwhile, the transposable element category and transport category are under-represented with *P* < 9.9e-10 and *P* < 9.1e-5, respectively. In total, 18 known miRNA families and 2 new miRNAs were predicted to target transcription factors or genes involved in regulation of transcription, suggesting that they have roles in post-transcriptional regulation.

### Validation of miRNA expression and anti-correlation with target transcripts

**Fig. 6:**
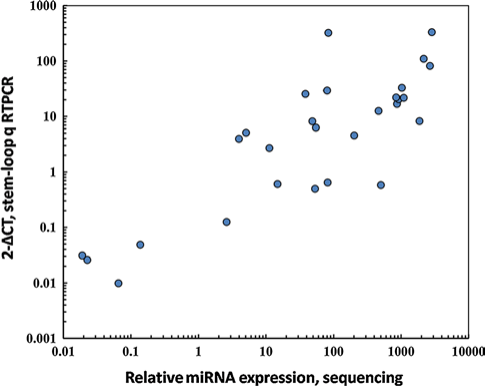
Comparison of deep sequencing data and stem–loop RT-PCR for measuring relative miRNA expression. Both the sequencing data and stem-loop RT-PCR were normalized against the internal control PASR17041531. A significant correlation was observed between the two methods (*ρ* = 0.78, *P* < 0.0001)

The deep sequencing data were validated using stem-loop real time RT-PCR to determine the expression profiles of seven conserved miRNAs (miR156, 159, 162, 167, 399, 528, 535) in four tissues (Supplemental Figure 7). These miRNAs were chosen because they represented a wide range of expression levels and differential tissue expression patterns. The stem-loop real time RT-PCR results were consistent with our deep sequencing data. The same differential expression patterns were also observed across all four libraries. A significant correlation (*ρ* = 0.78, *P* < 0.0001) was found between the deep sequencing data and the stem-loop real time RT-PCR measurements from technical replicates (Fig. 6). The correlation was also significant (*ρ* = 0.714, *P* < 0.01) in leaf and root libraries with three biological replicates. However, deep sequencing provided not only sequence information but also absolute read counts, and the detection of miRNA by deep sequencing was possible even at expression levels near the detection limits of real time RT-PCR.

PATC148826 (SPL protein), PATC140870 (Dicer-like protein), and PATC134326 (auxin response factor) are targets of miR156, miR162, and miR167, respectively. The abundance of these three target genes was investigated by quantitative real time RT-PCR (Supplemental Figure 7). The expression of miR162 was anti-correlated with that of its target (*ρ* = −1, *P* = 0.083). No significant correlation was seen between the expression levels of miR156 and miR167 and their target genes. This could be due to non-cleavage repression, feed-back regulation, spatial or temporal exclusion of miRNAs and their targets, the expression of other miRNAs leads to different levels of target repression, other levels of regulation exist such as promoter methylation, translational repression (Brodersen et al. 2008; Lanet et al. 2009), or because their relationships differ in different tissues. The RT-PCR is measuring the steady state level of RNA while the regulation of miRNA on targets is dynamic and changes under different circumstances. Further work is required to explore the regulatory mechanisms of various miRNAs and their targets in orchid.

### The existence of trans-acting siRNA in *P. aphrodite*

Trans-acting siRNA (ta-siRNA) activity has been found in Arabidopsis (Peragine et al. 2004; Vazquez et al. 2004). So far, four families of tasiRNA-generating TAS genes have been reported in Arabidopsis: TAS1 and TAS2 that are targeted by miR173, TAS3 that is targeted by miR390, and TAS4 that is targeted by miR828. By searching Orchidstra database, we identified several *P. aphrodite* transcripts that code for key components required for ta-siRNA biosynthesis, such as RNA-dependent RNA polymerase RDR6 (PATC131836), DCL4 (PATC128821, PATC150652), ARGONAUTE 7 (PATC068542), and suppressor of gene silencing 3 (PATC136902), indicating the operation of the ta-siRNA pathway in *P. aphrodite*. MiR390 was identified, and TAS3-derived tasiR-ARFs were also present in the flower, leaf, and seed libraries (PASR15422771 and PASR01102164). In addition, an *P. aphrodite* TAS3 homolog (PATC148096) that contains two target sites for miR390 flanking the tasiR-ARF producing regions (Supplemental Figure 8) was found in the Orchidstra database. The ta-siRNA biosynthesis pathway seems to be conserved in Orchids. Other TAS families, however, were not identified in this study despite the fact that the depth of sequencing achieved allowed the detection of extremely rare transcripts.

## Conclusions

In this study, systematic computational approaches were used to profile the small RNA transcriptome of the non-model plant *P. aphrodite* from deep sequencing data sets. A bioinformatics pipeline was established that allowed the characterization of expression profiles of orchid miRNAs from 88 miRNA families, and the identification of 23 new miRNAs, 91 miRNA precursors and 240 miRNA targets. Cleavage of predicted target transcripts was confirmed for selected miRNAs, including the new miRNA PA-miR1. tasiR-ARF and TAS3 transcript were also discovered, suggesting that the ta-siRNA pathway operates in orchid. All the known *P. aphrodite* miRNAs and predicted target genes in this study have been made freely available in the web-based orchid database Orchidstra. The comprehensive profiling of the *P. aphrodite* miRNAome achieved provides a useful reference for further investigation of miRNA in Orchidaceae. The systematic, integrated bioinformatics analysis pipeline developed will also be useful for analysis of the miRNAome from deep sequencing data of other non-model plants lacking a reference genome.

## Electronic supplementary material

Below is the link to the electronic supplementary material.

Search procedure for *P. aphrodite* miRNA precursors (TIFF 3829 kb)

Schematic representation of the bioinformatics pipeline for miRNA target prediction. miR166 is used as an example to demonstrate the approach. (a) To find the target of miRNA, the mature miRNA sequence (PASR12301454 and PASR12833208, annotated as miR166) was searched against protein-coding ESTs in the Orchidstra database by BLASTN with ungapped alignment. To filter miRNA target candidates, the resulting complementary alignment was checked for number of mismatches and length. Orchidstra protein-coding ESTs were also searched for homologous Arabidopsis targets using BLASTX. (b) An example of an identified target site. Both methods identified PATC146998 as a potential target of miR166. The complementary sites within the PATC146998 miR166 target are conserved in Arabidopsis (JPEG 549 kb)

The 88 known miRNA identified in this study and their distribution across plant species. The miRNA data are from this study, (An et al. 2011) and from the miRBase. The red blocks indicate the known miRNA families that were identified in both this study and (An et al. 2011), the blue blocks indicate the known miRNA families that were identified in this study but not found in (An et al. 2011). The plant species shown in this figure are *Chlamydomonas reinhardtii* (cre), *Pinus taeda* (pta), *Picea abies* (pab), *Pinus densata* (pde), *Physcomitrella patens* (ppt), *Selaginella moellendorffii* (smo), *Cynara cardunculus* (cca), *Helianthus annuus* (han), *Helianthus ciliaris* (hci), *Helianthus tuberosus* (htu), *Helianthus exilis* (hex), *Helianthus argophyllus* (har), *Helianthus petiolaris* (hpe), *Helianthus paradoxus* (hpa), *Arabidopsis thaliana* (ath), *Brassica napus* (bna), *Brassica oleracea* (bol), *Brassica rapa* (bra), *Arabidopsis lyrata* (aly), *Carica papaya* (cpa), *Cucumis melo* (cme), *Ricinus communis* (rco), *Manihot esculenta* (mes), *Hevea brasiliensis* (hbr), *Glycine max* (gma), *Lotus japonicus* (lja), *Medicago truncatula* (mtr), *Vigna unguiculata* (vun), *Phaseolus vulgaris* (pvu), *Arachis hypogaea* (ahy), *Glycine soja* (gso), *Acacia mangium* (amg), *Acacia auriculiformis* (aau), *Rehmannia glutinosa* (rgl), *Salvia sclarea* (ssl), *Digitalis purpurea* (dpr), *Gossypium herbaceum* (ghb), *Gossypium hirsutum* (ghr), *Gossypium raimondii* (gra), *Gossypium arboreum* (gar), *Theobroma cacao* (tcc), *Aquilegia caerulea* (aqc), *Bruguiera gymnorhiza* (bgy), *Bruguiera cylindrica* (bcy), *Malus domestica* (mdm), *Citrus sinensis* (csi), *Citrus clementine* (ccl), *Citrus reticulata* (crt), *Citrus trifoliata* (ctr), Populus trichocarpa (ptc), Populus euphratica (peu), *Solanum lycopersicum* (sly), *Nicotiana tabacum* (nta), *Solanum tuberosum* (stu), *Vitis vinifera* (vvi), *Phalaenopsis aphrodite* (pap) *Oryza sativa* (osa), *Sorghum bicolor* (sbi), *Saccharum officinarum* (sof), *Triticum aestivum* (tae), *Zea mays* (zma), *Brachypodium distachyon* (bdi), *Triticum turgidum* (ttu), *Aegilops tauschii* (ata), *Hordeum vulgare* (hvu), *Festuca arundinacea* (far), *Saccharum ssp.* (ssp), and *Elaeis guineensis* (egu) (JPEG 1135 kb)

An example of a *P. aphrodite* microRNA stem-loop structure. (a) Alignment of the miR166* sequence (PASR12908326) from *P. aphrodite* with homologs in *Zea mays* (zma-miR166a* and zma-miR166 m*). (b) The stem-loop structure of the precursor of miR166. The mature miRNA is marked by filled orange circles, and the miRNA* is marked by filled purple circles. This plot was generated by the Mfold program. (c) Alignment of mature miR166 (PASR12301454) from *P. aphrodite* with *Zea mays* (zma-miR166a and zma-miR166 m), *Oryza sativa* (osa-miR166a), and *Arabidopsis thaliana* (ath-miR166a) homologs (JPEG 301 kb)

Stem-loop structures of new miRNA precursors in *P. aphrodite*. Filled purple circles and filled orange circles indicate the mature miRNA derived from the 5′ arm and 3′ arm of the precursor miRNA, respectively (PDF 346 kb)

Screenshot of the main features of miRNA resource in Orchidstra. Our study generated a searchable online resource of orchid miRNAs, which has been integrated with the Orchidstra database. (a) The miRNA details page shows the ID, family, sequence, expression level, targets and precursors. A single-headed arrow denotes the page of corresponding data, while a double-headed arrow means reciprocal links between miRNA/target/precursor details pages. (b) The page for the expression levels of miRNA in each tissue. (c) The target details page shows the functional annotations and expression profile of target genes, and also provides link to the corresponding miRNA. (d) The precursor details page shows the sequence, structure information, and mapping results. Link to the corresponding miRNA is provided. (e) The page for the predicted stem-loop secondary structure of miRNA precursor and the positions of the mature miRNA. (f) The small RNA reads from deep sequencing were mapped to the precursors and the mapping results can be viewed in Orchidstra database (JPEG 1110 kb)

Quantitative RT-PCR for expression profiling of a subset of mature miRNAs and target genes in different tissues of *P. aphrodite*. The expression of miRNA and target gene were presented as the fold change after normalization to the internal control (PASR17041531) and actin gene, respectively. The grey bars represent the average fold change ± SE of three technical replicates. PATC148826, Squamosa promoter-binding-like protein, was targeted by miR156. PATC140870, Dicer-like protein 1, was targeted by miR162. PATC134326, auxin response factor 6, was targeted by miR167 (PDF 145 kb)

Multiple alignment of TAS3 transcripts from *P. aphrodite* and 14 other plant species. (a) The alignment of PaTAS3a in *P. aphrodite* (PATC148096) and its homologs of 14 other plant species, including *Panicum hallii* (JR068084), rice (OsTAS3a on chromosome 3), Arabidopsis (AT3G17185), *Malus* x *domestica* (CN490861), *Arachis hypogaea* (ES759435), *Manihot esculenta* (CK643507), *Citrus sinensis* (EY674848), *Quercus robur* (FP032778), *Lotus japonicus* (AK338955), *Glycine max* (CX710282), *Vigna unguiculata* (FG899643), *Vitis vinifera* (CF214752), *Theobroma cacao* (CU579872), *Gossypium hirsutum* (DW503626). Multiple alignment analyses were performed using MUSCLE and edited using Jalview. (b) Highly conserved sequences in the regions of miR390 target sites and tasiRNA-ARF sites (PDF 519 kb)

List of primer sequences used in this study (PDF 10 kb)

Adapter and primers used for 5′ RACE experiments (PDF 7 kb)

## Abbreviations

ASRP: The Arabidopsis small RNA project
Ct: Threshold cycle
DAS: Days after sowing
DCL1: Dicer-like 1
DGE: Digital gene expression profiling
EST: Expressed sequence tags
GAMYB: Gibberellin (GA)-inducible MYB-transcription factor
GO: Gene ontology
HOX: Homeobox-leucine zipper protein HOX32
HYL1: Hyponastic Leaves 1
MFE: Minimum free energy
miRBase: The microRNA database
NGS: Next generation sequencing
ρ: Spearman’s rank correlation coefficient
RLM-5′ RACE: RNA ligase-mediated rapid amplification of 5′ ends
RT-PCR: Reverse transcriptase-polymerase chain reaction
RPM: Reads per million
snRNA: Small nuclear RNA
snoRNA: Small nucleolar RNA
SPL: SQUAMOSA promoter-binding-like
SPM: Specificity measure
TAIR: The Arabidopsis information resource
ta-siRNA: Trans-acting siRNA
