# Supplementary figures and images for: Identification and characterization of the microRNA transcriptome of a moth orchid Phalaenopsis aphrodite
Source: Plant Mol Biol. 2013 Oct 31;84(4):529–48. doi: 10.1007/s11103-013-0150-0 (PMC3920020; doi:10.1007/s11103-013-0150-0)

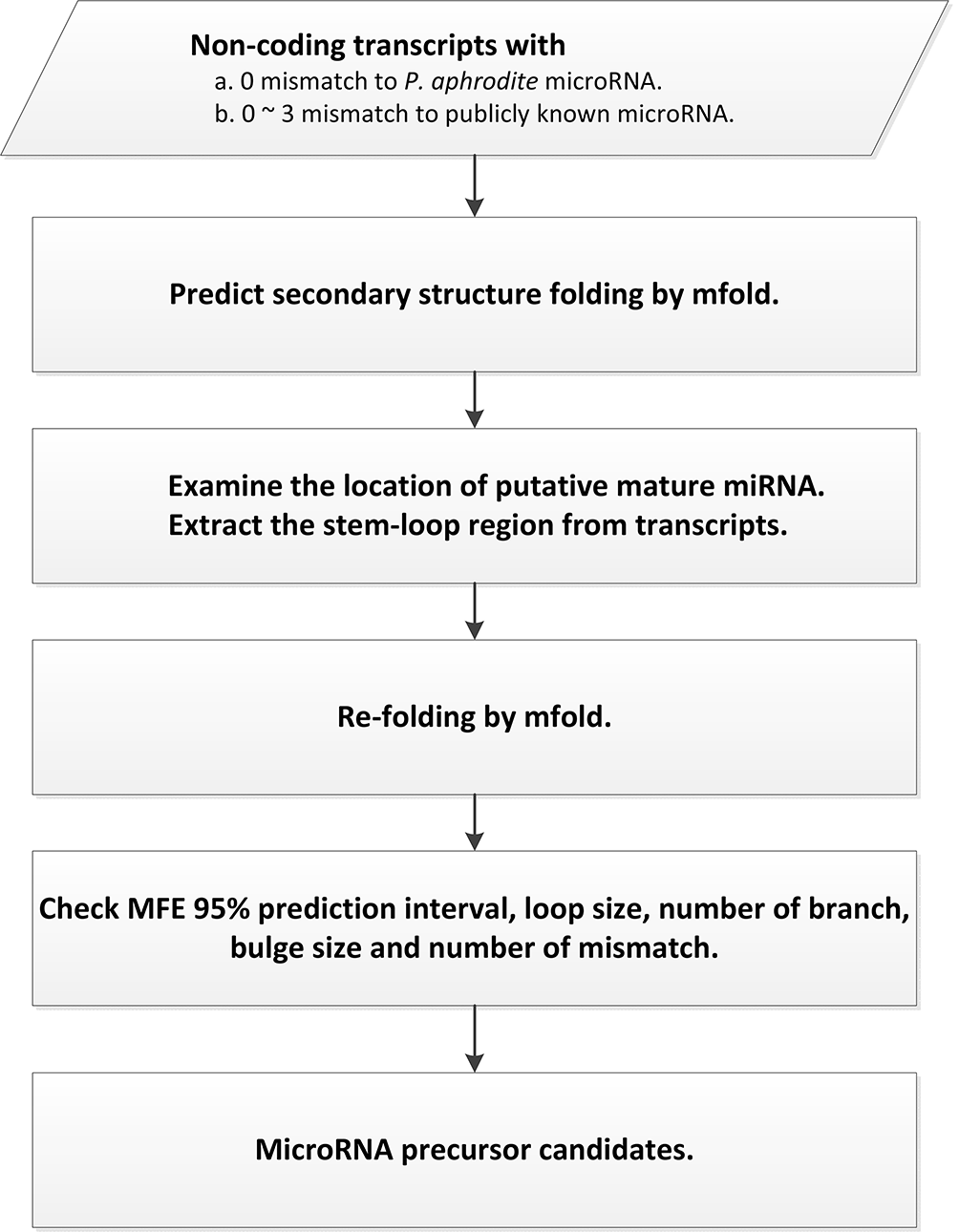

Supplement: Supplementary file 1 — Search procedure for P. aphrodite miRNA precursors (TIFF 3829 kb) [file 11103_2013_150_MOESM1_ESM.tif]

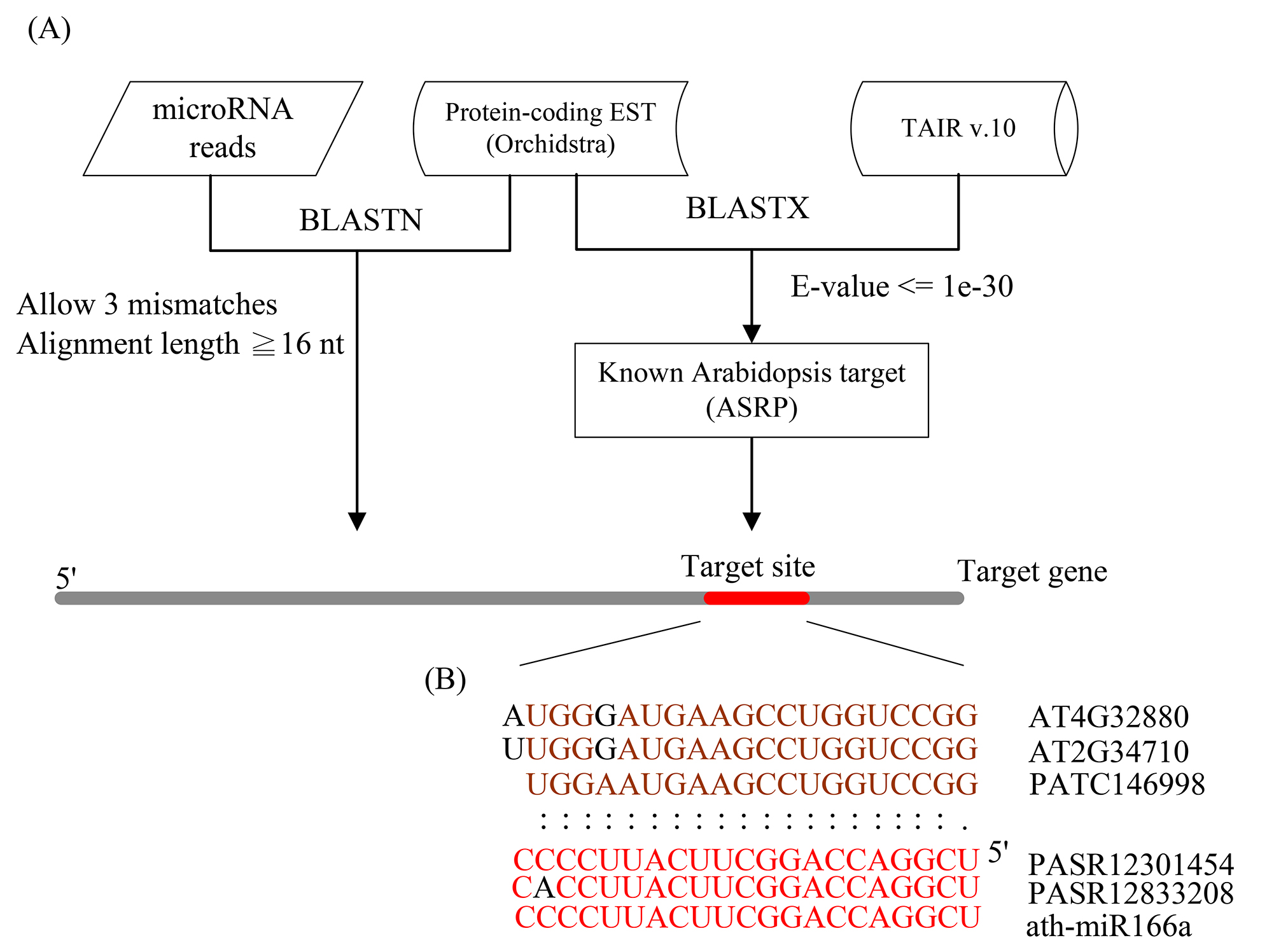

Supplement: Supplementary file 2 — Schematic representation of the bioinformatics pipeline for miRNA target prediction. miR166 is used as an example to demonstrate the approach. (a) To find the target of miRNA, the mature miRNA sequence (PASR12301454 and PASR12833208, annotated as miR166) was searched against protein-coding ESTs in the Orchidstra database by BLASTN with ungapped alignment. To filter miRNA target candidates, the resulting complementary alignment was checked for number of mismatches and length. Orchidstra protein-coding ESTs were also searched for homologous Arabidopsis targets using BLASTX. (b) An example of an identified target site. Both methods identified PATC146998 as a potential target of miR166. The complementary sites within the PATC146998 miR166 target are conserved in Arabidopsis (JPEG 549 kb) [file 11103_2013_150_MOESM2_ESM.jpg]

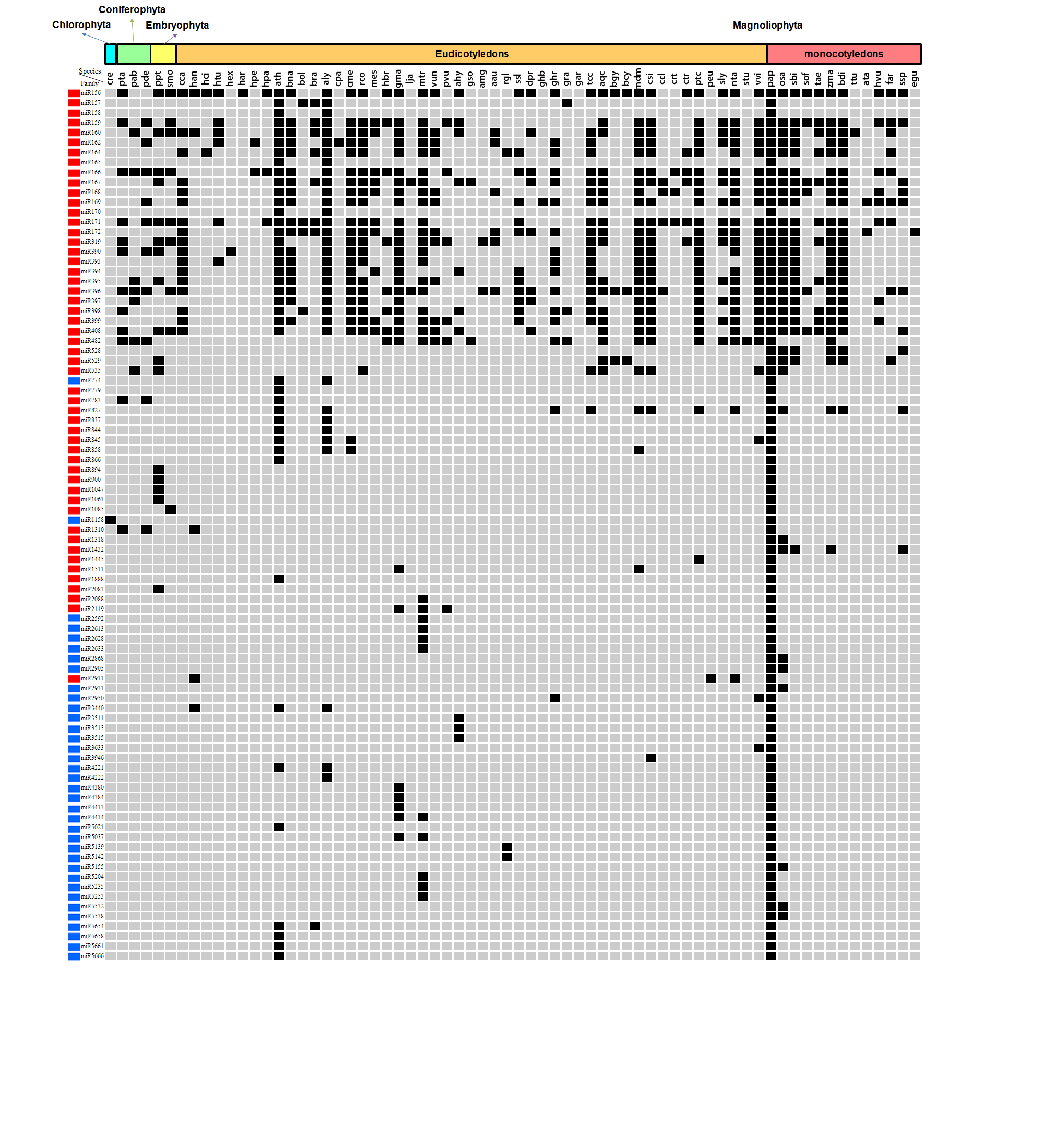

Supplement: Supplementary file 3 — The 88 known miRNA identified in this study and their distribution across plant species. The miRNA data are from this study, (An et al. 2011) and from the miRBase. The red blocks indicate the known miRNA families that were identified in both this study and (An et al. 2011), the blue blocks indicate the known miRNA families that were identified in this study but not found in (An et al. 2011). The plant species shown in this figure are Chlamydomonas reinhardtii (cre), Pinus taeda (pta), Picea abies (pab), Pinus densata (pde), Physcomitrella patens (ppt), Selaginella moellendorffii (smo), Cynara cardunculus (cca), Helianthus annuus (han), Helianthus ciliaris (hci), Helianthus tuberosus (htu), Helianthus exilis (hex), Helianthus argophyllus (har), Helianthus petiolaris (hpe), Helianthus paradoxus (hpa), Arabidopsis thaliana (ath), Brassica napus (bna), Brassica oleracea (bol), Brassica rapa (bra), Arabidopsis lyrata (aly), Carica papaya (cpa), Cucumis melo (cme), Ricinus communis (rco), Manihot esculenta (mes), Hevea brasiliensis (hbr), Glycine max (gma), Lotus japonicus (lja), Medicago truncatula (mtr), Vigna unguiculata (vun), Phaseolus vulgaris (pvu), Arachis hypogaea (ahy), Glycine soja (gso), Acacia mangium (amg), Acacia auriculiformis (aau), Rehmannia glutinosa (rgl), Salvia sclarea (ssl), Digitalis purpurea (dpr), Gossypium herbaceum (ghb), Gossypium hirsutum (ghr), Gossypium raimondii (gra), Gossypium arboreum (gar), Theobroma cacao (tcc), Aquilegia caerulea (aqc), Bruguiera gymnorhiza (bgy), Bruguiera cylindrica (bcy), Malus domestica (mdm), Citrus sinensis (csi), Citrus clementine (ccl), Citrus reticulata (crt), Citrus trifoliata (ctr), Populus trichocarpa (ptc), Populus euphratica (peu), Solanum lycopersicum (sly), Nicotiana tabacum (nta), Solanum tuberosum (stu), Vitis vinifera (vvi), Phalaenopsis aphrodite (pap) Oryza sativa (osa), Sorghum bicolor (sbi), Saccharum officinarum (sof), Triticum aestivum (tae), Zea mays (zma), Brachypodium distachyon (bdi), Tri [file 11103_2013_150_MOESM3_ESM.jpg]

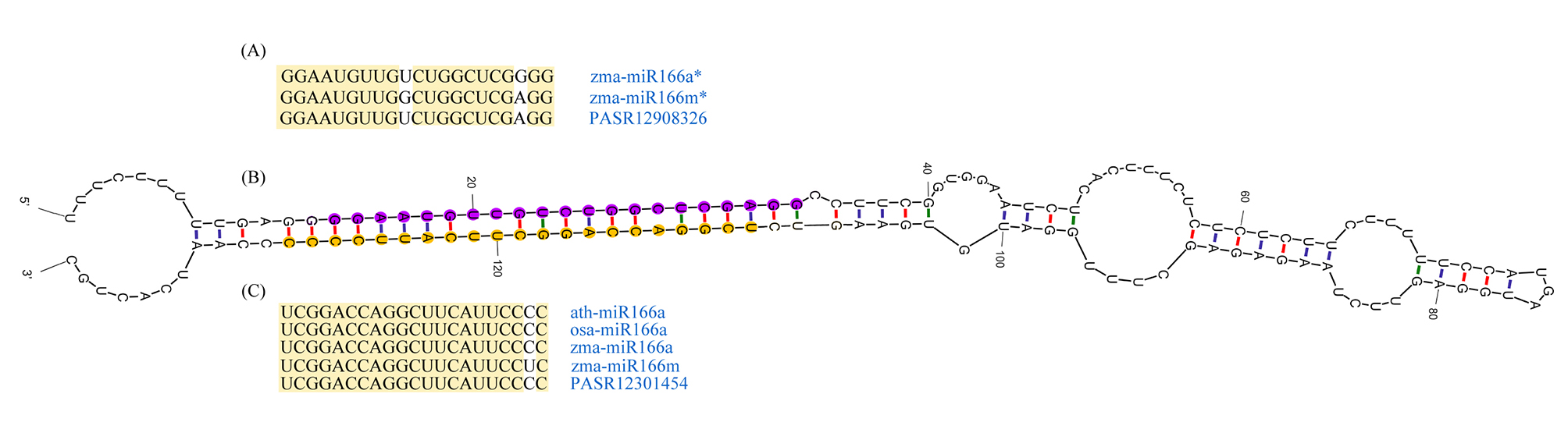

Supplement: Supplementary file 4 — An example of a P. aphrodite microRNA stem-loop structure. (a) Alignment of the miR166* sequence (PASR12908326) from P. aphrodite with homologs in Zea mays (zma-miR166a* and zma-miR166 m*). (b) The stem-loop structure of the precursor of miR166. The mature miRNA is marked by filled orange circles, and the miRNA* is marked by filled purple circles. This plot was generated by the Mfold program. (c) Alignment of mature miR166 (PASR12301454) from P. aphrodite with Zea mays (zma-miR166a and zma-miR166 m), Oryza sativa (osa-miR166a), and Arabidopsis thaliana (ath-miR166a) homologs (JPEG 301 kb) [file 11103_2013_150_MOESM4_ESM.jpg]

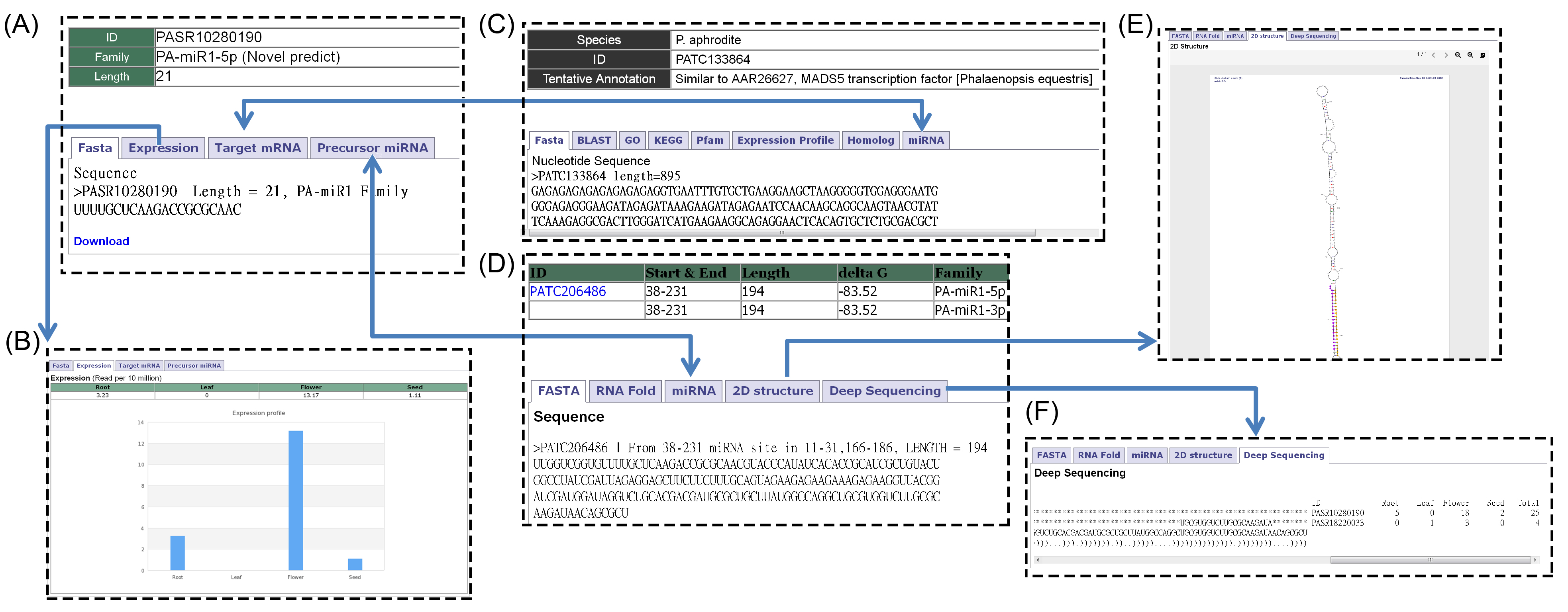

Supplement: Supplementary file 6 — Screenshot of the main features of miRNA resource in Orchidstra. Our study generated a searchable online resource of orchid miRNAs, which has been integrated with the Orchidstra database. (a) The miRNA details page shows the ID, family, sequence, expression level, targets and precursors. A single-headed arrow denotes the page of corresponding data, while a double-headed arrow means reciprocal links between miRNA/target/precursor details pages. (b) The page for the expression levels of miRNA in each tissue. (c) The target details page shows the functional annotations and expression profile of target genes, and also provides link to the corresponding miRNA. (d) The precursor details page shows the sequence, structure information, and mapping results. Link to the corresponding miRNA is provided. (e) The page for the predicted stem-loop secondary structure of miRNA precursor and the positions of the mature miRNA. (f) The small RNA reads from deep sequencing were mapped to the precursors and the mapping results can be viewed in Orchidstra database (JPEG 1110 kb) [file 11103_2013_150_MOESM6_ESM.jpg]

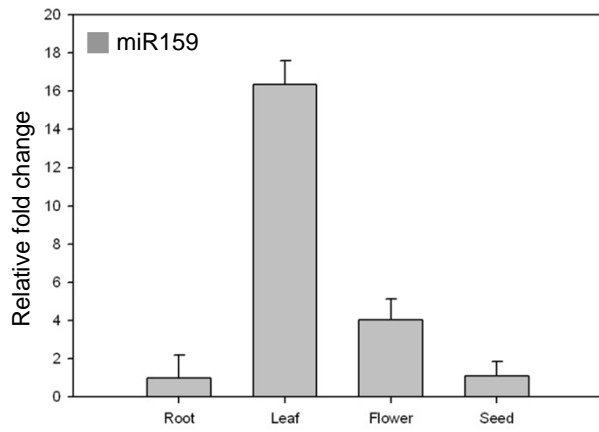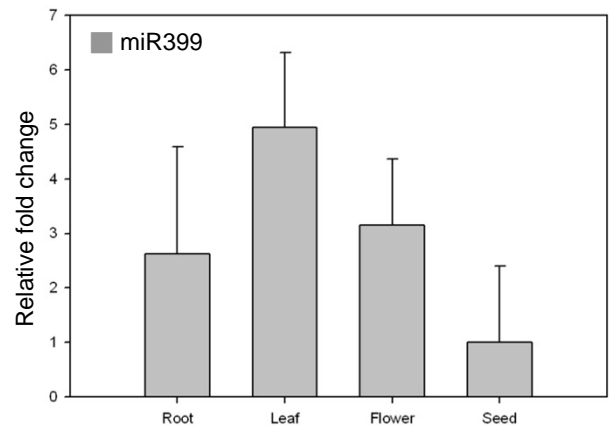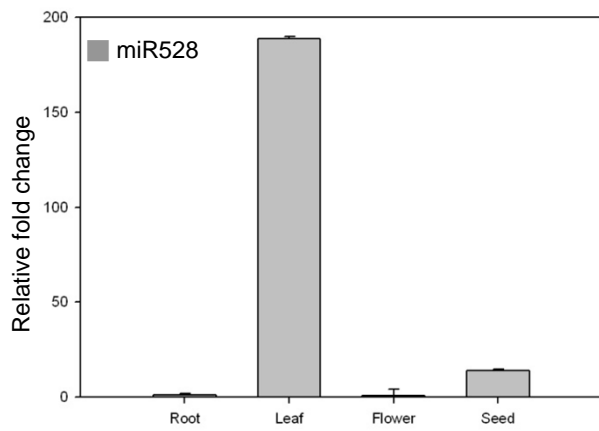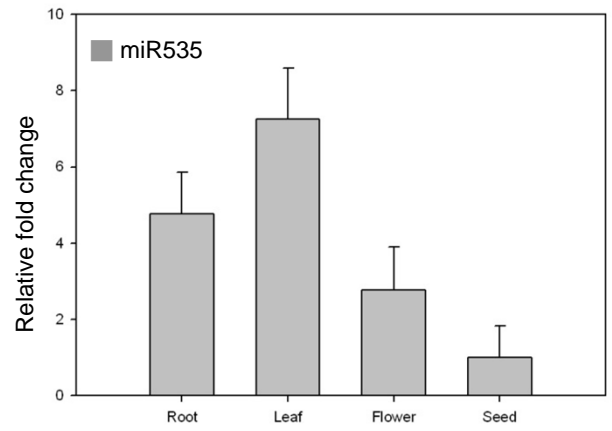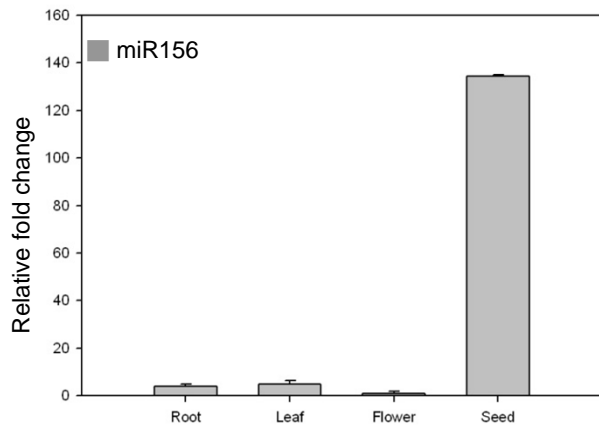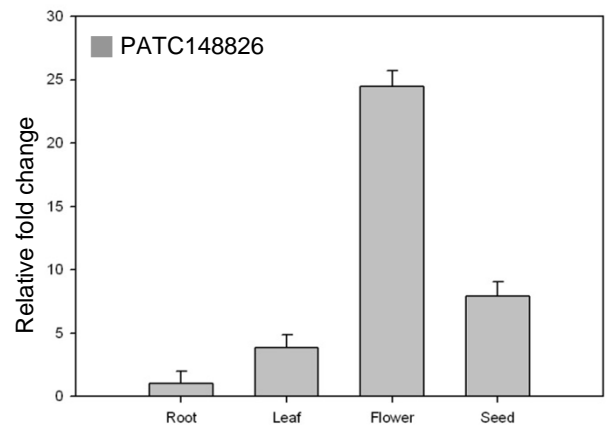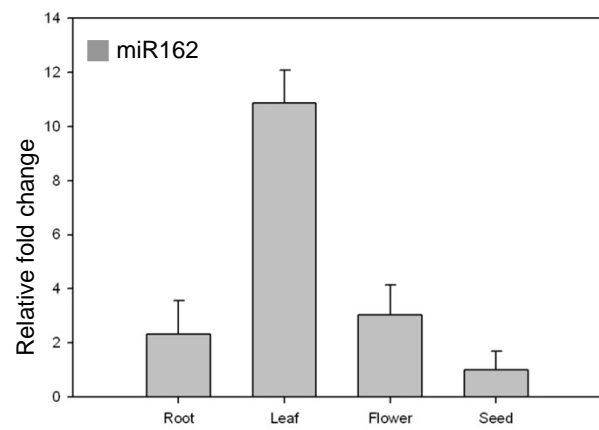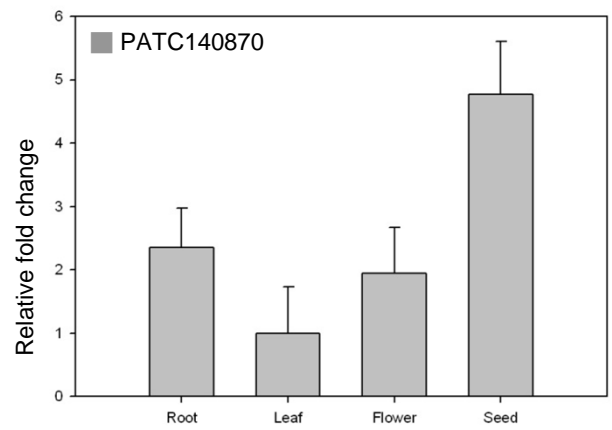

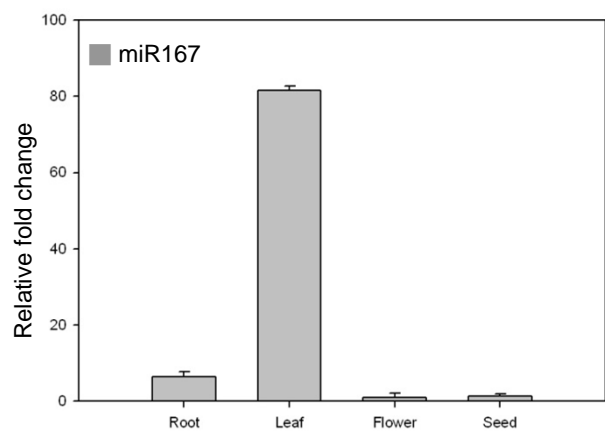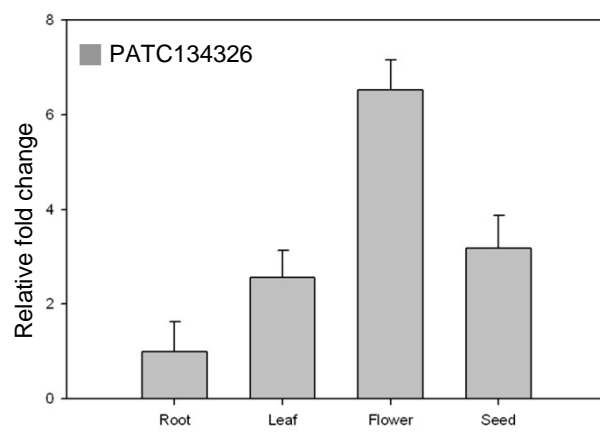

Supplement: Supplementary file 7 — Quantitative RT-PCR for expression profiling of a subset of mature miRNAs and target genes in different tissues of P. aphrodite. The expression of miRNA and target gene were presented as the fold change after normalization to the internal control (PASR17041531) and actin gene, respectively. The grey bars represent the average fold change ± SE of three technical replicates. PATC148826, Squamosa promoter-binding-like protein, was targeted by miR156. PATC140870, Dicer-like protein 1, was targeted by miR162. PATC134326, auxin response factor 6, was targeted by miR167 (PDF 145 kb) [file 11103_2013_150_MOESM7_ESM.pdf]
