# Supplementary material for: Identification and characterization of the microRNA transcriptome of a moth orchid Phalaenopsis aphrodite
Source: Plant Mol Biol. 2013 Oct 31;84(4):529–48. doi: 10.1007/s11103-013-0150-0 (PMC3920020; doi:10.1007/s11103-013-0150-0)
Supplement: Supplementary file 5 — Stem-loop structures of new miRNA precursors in P. aphrodite. Filled purple circles and filled orange circles indicate the mature miRNA derived from the 5′ arm and 3′ arm of the precursor miRNA, respectively (PDF 346 kb) [file 11103_2013_150_MOESM5_ESM.pdf]

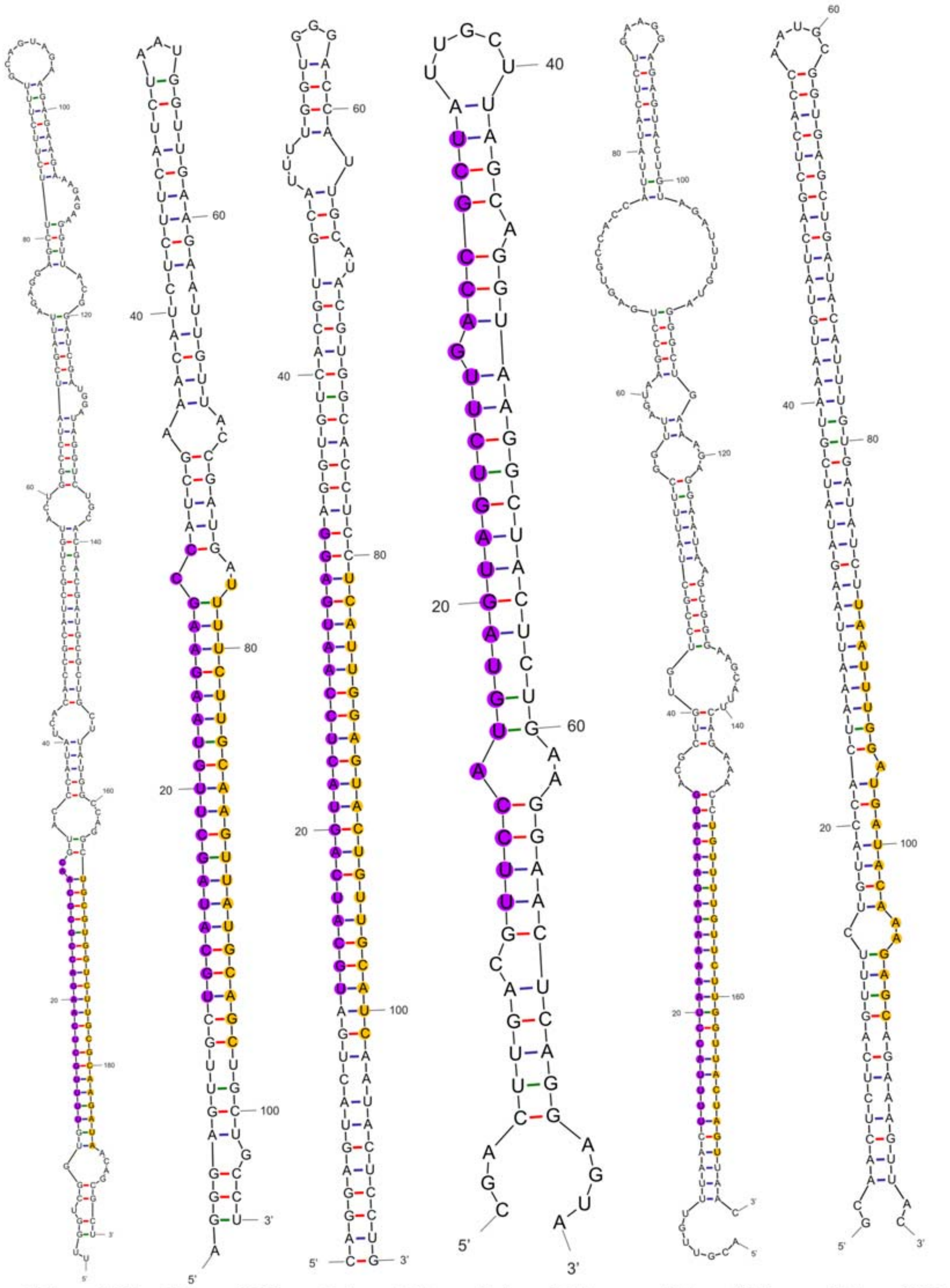

PA-miR1 PA-miR2 PA-miR3 PA-miR4 PA-miR5 PA-miR6

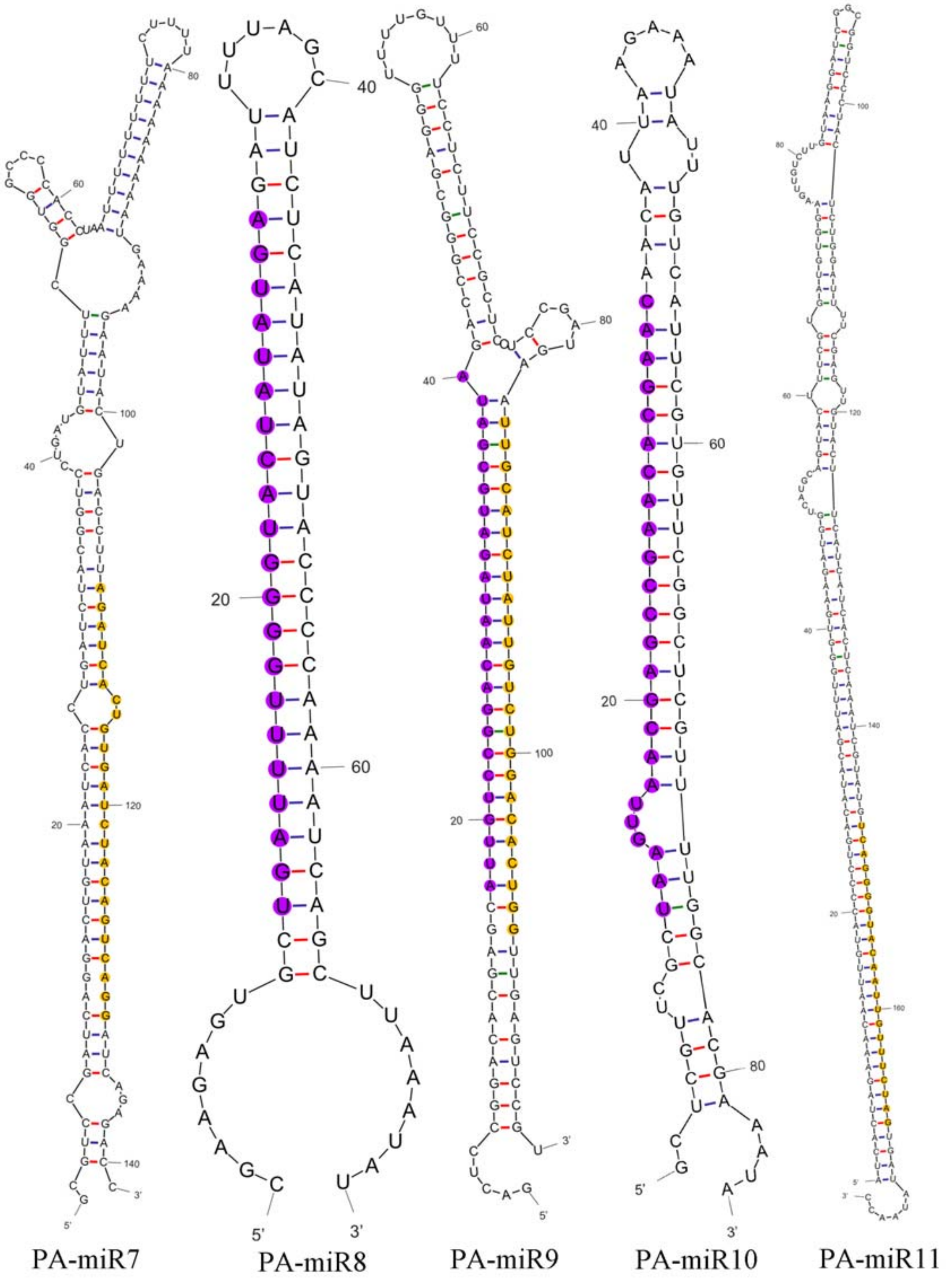
PA-miR7
PA-miR8
PA-miR9
PA-miR10
PA-miR11

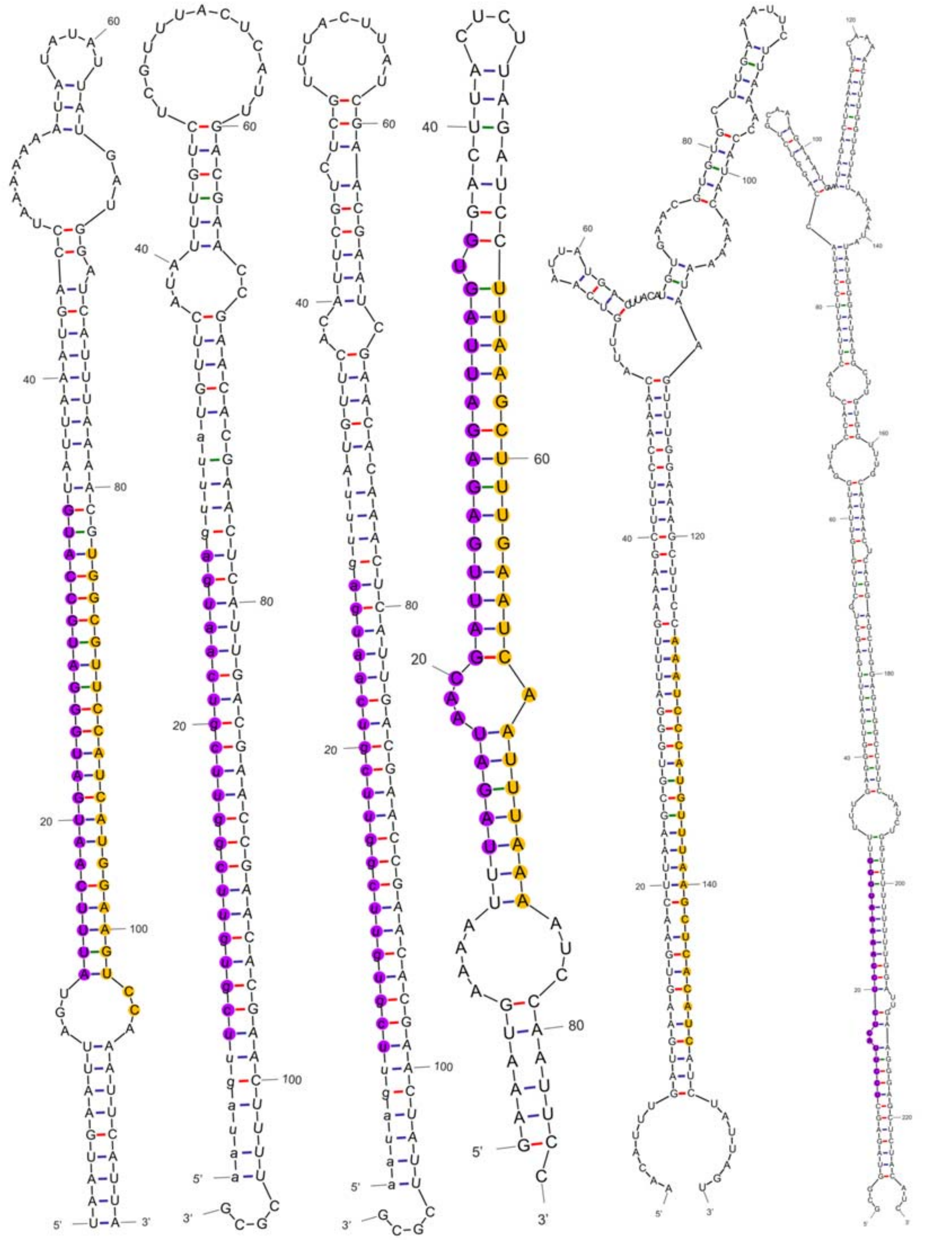

Supplemental Figure 5 Stem-loop structures of new miRNA precursors in *P. aphrodite*
