# Supplementary material for: Identification and characterization of the microRNA transcriptome of a moth orchid Phalaenopsis aphrodite
Source: Plant Mol Biol. 2013 Oct 31;84(4):529–48. doi: 10.1007/s11103-013-0150-0 (PMC3920020; doi:10.1007/s11103-013-0150-0)
Supplement: Supplementary file 8 — Multiple alignment of TAS3 transcripts from P. aphrodite and 14 other plant species. (a) The alignment of PaTAS3a in P. aphrodite (PATC148096) and its homologs of 14 other plant species, including Panicum hallii (JR068084), rice (OsTAS3a on chromosome 3), Arabidopsis (AT3G17185), Malus x domestica (CN490861), Arachis hypogaea (ES759435), Manihot esculenta (CK643507), Citrus sinensis (EY674848), Quercus robur (FP032778), Lotus japonicus (AK338955), Glycine max (CX710282), Vigna unguiculata (FG899643), Vitis vinifera (CF214752), Theobroma cacao (CU579872), Gossypium hirsutum (DW503626). Multiple alignment analyses were performed using MUSCLE and edited using Jalview. (b) Highly conserved sequences in the regions of miR390 target sites and tasiRNA-ARF sites (PDF 519 kb) [file 11103_2013_150_MOESM8_ESM.pdf]

Supplemental Figure 8 Multiple alignment of TAS3 transcripts from *P. aphrodite* and 14 other plant species.

(a) The alignment of PaTAS3a in *P. aphrodite* (PATC148096) and its homologs of 14 other plant species, including *Panicum hallii* (JR068084), rice (OsTAS3a on chromosome 3) (Wang et al. 2010), Arabidopsis (AT3G17185), *Malus* x *domestica* (CN490861) (Xia et al. 2012), *Arachis hypogaea* (ES759435), *Manihot esculenta* (CK643507), *Citrus sinensis* (EY674848), *Quercus robur* (FP032778), *Lotus japonicus* (AK338955), *Glycine max* (CX710282), *Vigna unguiculata* (FG899643), *Vitis vinifera* (CF214752), *Theobroma cacao* (CU579872), *Gossypium hirsutum* (DW503626). Multiple alignment analyses were performed using MUSCLE (Edgar 2004) and edited using Jalview (Waterhouse et al. 2009). (b) Highly conserved sequences in the regions of miR390 target sites and tasiRNA-ARF sites.

References

Edgar RC (2004) MUSCLE: multiple sequence alignment with high accuracy and high throughput. Nucleic acids research 32 (5):1792-1797. doi:10.1093/nar/gkh340

Wang J, Gao X, Li L, Shi X, Zhang J, Shi Z (2010) Overexpression of Osta-siR2141 caused abnormal polarity establishment and retarded growth in rice. Journal of experimental botany 61 (6):1885-1895. doi:10.1093/jxb/erp378

Waterhouse AM, Procter JB, Martin DM, Clamp M, Barton GJ (2009) Jalview Version 2--a multiple sequence alignment editor and analysis workbench. Bioinformatics 25 (9):1189-1191. doi:10.1093/bioinformatics/btp033

Xia R, Zhu H, An YQ, Beers EP, Liu Z (2012) Apple miRNAs and tasiRNAs with novel regulatory networks. Genome biology 13 (6):R47. doi:10.1186/gb-2012-13-6-r47

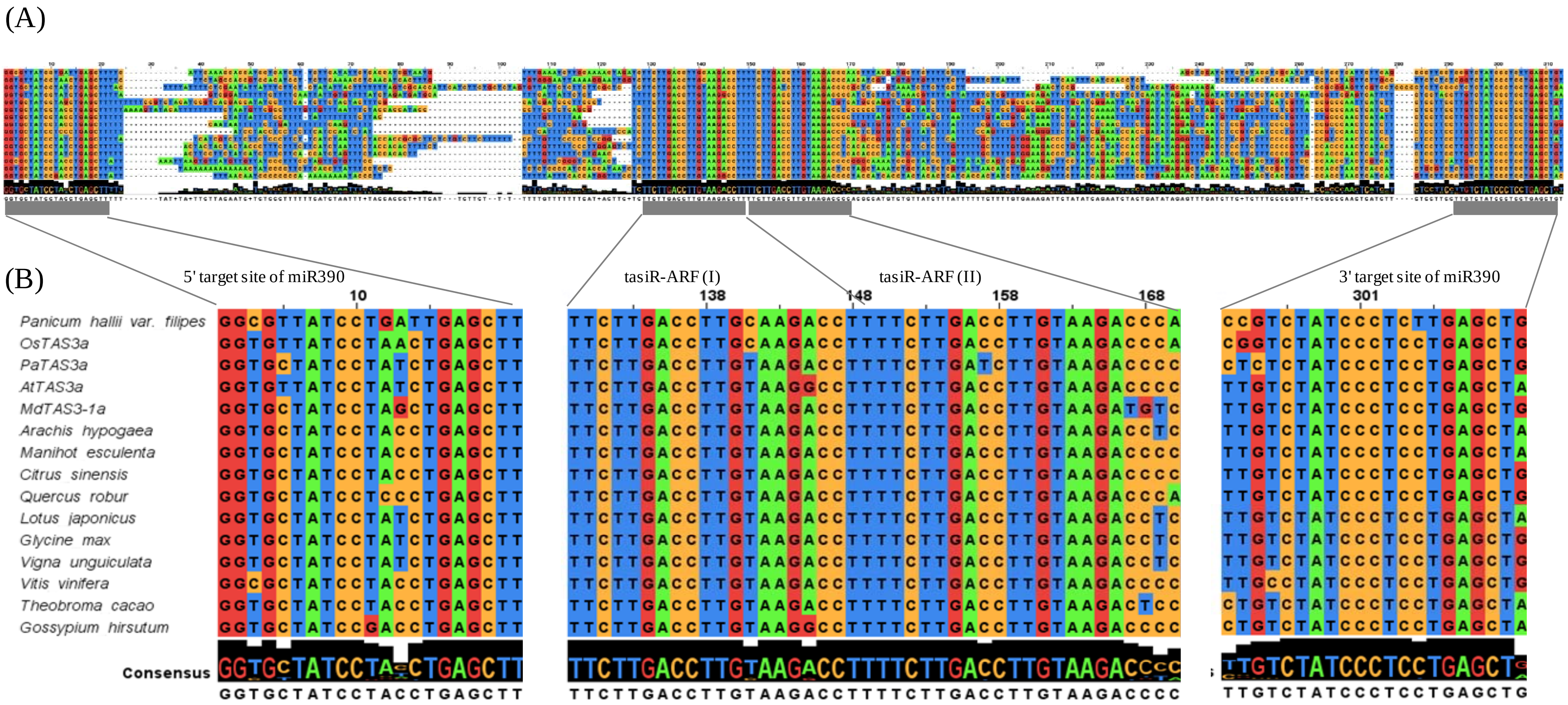

(A)
(B)
5' target site of miRNA390
tasiR-ARF (I)
tasiR-ARF (II)
3' target site of miR390
Panicum hallii var. filipes
OsTAS3a
PaTAS3a
AtTAS3a
MdTAS3-1a
Arachis hypogaea
Manihot esculenta
Citrus sinensis
Quercus robur
Lotus japonicus
Glycine max
Vigna unguiculata
Vitis vinifera
Theobroma cacao
Gossypium hirsutum
Consensus
GGTGCTATCCTACCTGAGCTT
TTCTTGACCTTGTAAGACCTTTTCTTGACCTTGTAAGACCCC
TTGTCTATCCCTCCTGAGCTG
