# Supplementary material for: Identification and characterization of the microRNA transcriptome of a moth orchid Phalaenopsis aphrodite
Source: Plant Mol Biol. 2013 Oct 31;84(4):529–48. doi: 10.1007/s11103-013-0150-0 (PMC3920020; doi:10.1007/s11103-013-0150-0)
Supplement: Supplementary file 9 — List of primer sequences used in this study (PDF 10 kb) [file 11103_2013_150_MOESM9_ESM.pdf]

Supplemental Table 1 List of primer sequences used in this study

Family/Orchidstra ID	Stem-loop RT primer	Forward primer
miR156 / PASR13401425	5'-GTCGTATCCAGTGCAGGGTCCGA GGTATTCGCACTGGATACGACGTGC TC	5'-CGGGGTGACAGAAGAGAGT
miR159 / PASR02106156	5'-GTCGTATCCAGTGCAGGGTCCGAG GTATTCGCACTGGATACGACCAGAG C	5'-CGCGTTTGGATTGAAGGGA
miR162 / PASR17893510	5'-GTCGTATCCAGTGCAGGGTCCGAG GTATTCGCACTGGATACGACCCGGAT	5'-CCGCTCGATAAACCTCTGC
miR167 / PASR07290166	5'-GTCGTATCCAGTGCAGGGTCCGAG GTATTCGCACTGGATACGACTCAGAT	5'-GGATGAAGCTGCCAGCATG
miR399 / PASR13983513	5'-GTCGTATCCAGTGCAGGGTCCGAG GTATTCGCACTGGATACGACCAGGG C	5'-GGCGTGCCAAAGGAGAATT
miR528 / PASR12940280	5'-GTCGTATCCAGTGCAGGGTCCGAG GTATTCGCACTGGATACGACCTCCTC	5'-ACGTGGAAGTGGCATGCA
miR535 / PASR11244591	5'-GTCGTATCCAGTGCAGGGTCCGAG GTATTCGCACTGGATACGACGCGTGC	5'-CACGCATGACAACGAGAGAG
miR5139 / PASR 17041531	5'-GTCGTATCCAGTGCAGGGTCCGAG GTATTCGCACTGGATACGACTGGTAT	5'-GGCGCAACCTGGCTCTG
Orchidstra ID	Reverse primer	Forward primer
PATC148826 (Amplicon size: 131)	5'-CGGCGATGTTTCGGTATC (Tm = 60.03)	5'-CATCCGTCGGCTCTTCAT (Tm = 59.74)
PATC140870 (Amplicon size: 126)	5'-CAATGAAGCCATCGTCCC (Tm = 60.06)	5'-CGGCTTCGTATTGGTTCG (Tm = 60.21)
PATC134326 (Amplicon size: 108)	5'-GCGAAACTCCGATTCCCT (Tm = 60.16)	5'-ATGGCGGAAACATTGGTG (Tm = 60.33)
