# Supplementary material for: Identification and characterization of the microRNA transcriptome of a moth orchid Phalaenopsis aphrodite
Source: Plant Mol Biol. 2013 Oct 31;84(4):529–48. doi: 10.1007/s11103-013-0150-0 (PMC3920020; doi:10.1007/s11103-013-0150-0)
Supplement: Supplementary file 10 — Adapter and primers used for 5′ RACE experiments (PDF 7 kb) [file 11103_2013_150_MOESM10_ESM.pdf]

Supplemental Table 2 Adapter and primers used for 5' RACE experiments

Name	Sequence
RNA adapter	5'-CGACUGGAGCACGAGGACACUGACAUGGACUGAAGGAGUAGAAA-3'
RACE 5' primer	5'-CGACTGGAGCACGAGGACACTGA-3'
RACE 5' nested primer	5'-GGACACTGACATGGACTGAAGGAGTA-3'
PATC146998-GSP	5'-TGGCCTTAGAACCTCAGGCACACTT-3'
PATC152414-GSP	5'-GGCAATTGTCACATCCTCCACACCAT-3'
PATC140870-GSP	5'-GAATCAACCCCGCTGCCTTCACAAT-3'
PATC140870-nested GSP	5'-CGGATTACACCAAGCACGCGAAGAG-3'
PATC148783-GSP	5'-GCCCGACGGTGCAGCTATAAATTCA-3'
PATC148783-nested GSP	5'-TGTCCTGTTCCTCACCCAGCATTGT-3'
PATC138350-GSP	5'-GGCATACATGTGAGACCCGTCATTTGC-3'
PATC138350-nested GSP	5'-AGTTCGTGCATTAAGTTCCGGTGTGT-3'
